# Methionine Biosynthesis is Essential for Infection in the Rice Blast Fungus *Magnaporthe oryzae*

**DOI:** 10.1371/journal.pone.0111108

**Published:** 2015-04-09

**Authors:** Marie Emmanuelle Saint-Macary, Crystel Barbisan, Marie Josèphe Gagey, Océane Frelin, Roland Beffa, Marc Henri Lebrun, Michel Droux

**Affiliations:** 1 UMR 5240 MAP, UMR 5240 CNRS-UCB-INSA-BCS, Bayer CropScience, F-69263, Lyon, France; 2 Biochemistry Department, Bayer CropScience, F-69263, Lyon, France; Université Paris-Sud, FRANCE

## Abstract

Methionine is a sulfur amino acid standing at the crossroads of several biosynthetic pathways. In fungi, the last step of methionine biosynthesis is catalyzed by a cobalamine-independent methionine synthase (Met6, EC 2.1.1.14). In the present work, we studied the role of Met6 in the infection process of the rice blast fungus, *Magnaporthe oryzae*. To this end *MET6* null mutants were obtained by targeted gene replacement. On minimum medium, *MET6* null mutants were auxotrophic for methionine. Even when grown in presence of excess methionine, these mutants displayed developmental defects, such as reduced mycelium pigmentation, aerial hypha formation and sporulation. They also displayed characteristic metabolic signatures such as increased levels of cysteine, cystathionine, homocysteine, *S*-adenosylmethionine, *S*-adenosylhomocysteine while methionine and glutathione levels remained unchanged. These metabolic perturbations were associated with the over-expression of *MgCBS1* involved in the reversed transsulfuration pathway that metabolizes homocysteine into cysteine and *MgSAM1* and *MgSAHH1* involved in the methyl cycle. This suggests a physiological adaptation of *M*. *oryzae* to metabolic defects induced by the loss of Met6, in particular an increase in homocysteine levels. Pathogenicity assays showed that *MET6* null mutants were non-pathogenic on both barley and rice leaves. These mutants were defective in appressorium-mediated penetration and invasive infectious growth. These pathogenicity defects were rescued by addition of exogenous methionine and *S*-methylmethionine. These results show that *M*. *oryzae* cannot assimilate sufficient methionine from plant tissues and must synthesize this amino acid *de novo* to fulfill its sulfur amino acid requirement during infection.

## Introduction

The fungal species *Magnaporthe oryzae* is responsible of major diseases on cereals, including rice blast [1]. To infect its host plant, *M*. *oryzae* differentiates an appressorium on the leaf surface that mediates its penetration into host tissues [2]. Inside the plant cell, the fungus differentiates bulbous infectious hyphae in tight contact with the plant plasma membrane [3] that spread into adjacent host cells by puncturing plant cell walls. *M*. *oryzae* colonizes host tissues without causing major damage for 4–5 days (biotrophic phase). Then, the fungus rapidly destroys infected host tissues (necrotrophic phase) and produces conidiophores that release spores spreading the disease in rice fields [1]. Studies on *M*. *oryzae* infection has benefited from the availability of efficient molecular tools, including its genome sequence, leading to the identification of a large number of genes required for infection [1–6]. In particular these studies have highlighted the importance in infection of specific nutritional pathways such as lipid beta-oxydation, gluconeogenesis, trehalose cycle, purine and amino-acid biosynthesis, as well as NADPH and redox homeostasis [6].

**Fig 1:**
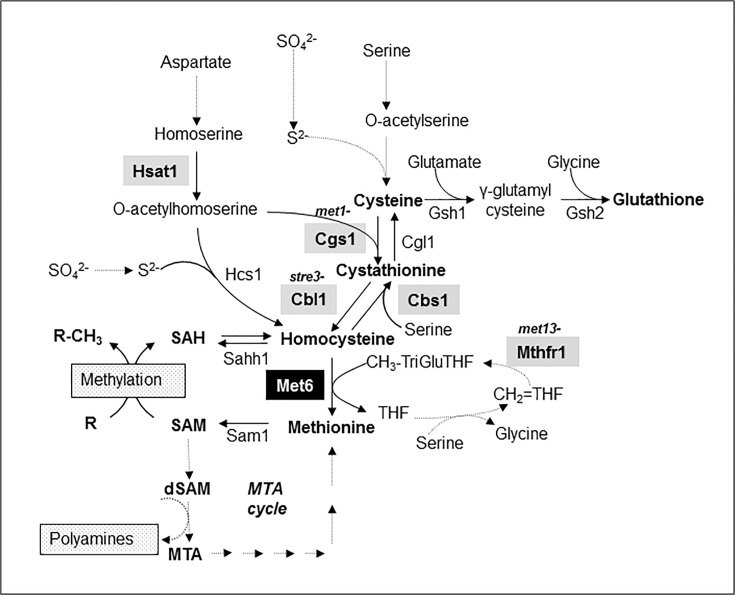
Sulfur amino acids biosynthetic pathway in filamentous fungi. Genes encoding enzymes from the transsulfuration pathway (cysteine to methionine, *CGS1*, *CBL1*), the reverse transsulfuration pathway (homocysteine to cysteine, *CBS1* and *CGL1*), the sulphydrylation pathway (*HSAT1*, *HCS1*), the methyl cycle (*MET6*, *SAM1*, *SAHH1*) and the glutathione biosynthesis pathway (*GSH1*, *GSH2*) were identified in *Magnaporthe oryzae* (v7) genome by similarity to known fungal genes. Proteins were designated as: **Cgs1**, Cystathionine γ-synthase (MGG_03583.7: ***met1-***); **Cbl1**, Cystathionine *β*-lyase (MGG_07074.7: ***str3-***); **Cbs1**, Cystathionine *β*-synthase (MGG_07384.7); **Cgl1**, Cystathionine γ-lyase (MGG_10380.7); **Hcs1**, Homocysteine synthase (MGG_07195.7); **Hsat1**, Homoserine acetyltransferase (MGG_01469.7); **Met6**, Methionine synthase (MGG_06712.7); **Mthfr1**, methylenetetrahydrofolate reductase (MGG_01728.7: ***met13-***); **Sahh1,** reversible *S*-adenosylhomocysteine hydrolase (MGG_05155.7); **Sam1**, *S*-adenosylmethionine synthetase (MGG_00383.7); **Gsh1**, γ-Glutamylcysteine synthetase (MGG_07317.7); **Gsh2**, Glutathione synthetase (MGG_06454.7). Abbreviations: CH_2_ = THF, methylene tetrahydrofolate; CH_3_-TriGluTHF, methyl tetrahydrofolate tri-glutamate; dSAM, desoxyribosyl-SAM; MTA, methylthioadenosine; R, compound to be methylated; R-CH_3_, methylated compound; SAM, *S*-adenosylmethionine; SAH, *S*-adenosylhomocysteine; SAM, *S*-adenosylmethionine; S^2-^, sulphur; SO_4_^2-^, sulphate; THF, tetrahydrofolate. Known fungal mutants are highlighted by grey boxes. Met6 is highlighted by a black box.

Nutritional strategies of a leaf fungal plant pathogen differ depending on the stage of infection. On the plant surface, *M*. *oryzae* uses nutrients stored in spores for germimation and appressorium differentiation [2, 6]. After penetration into host plant cells, *M*. *oryzae* infectious hyphae takes up and metabolizes sugar and nitrogen sources from the plant to support its growth and sporulation. However, some plant amino acids such as cysteine, methionine, tryptophan, histidine and arginine are only present in trace amounts in the leaf apoplast and are likely not available for fungal nutrition [6–8]. Infectious hyphae are expected to synthesize these amino acids from abundant apoplastic amino-acid such as glutamate or aspartate. Genetic studies in *M*. *oryzae* support this hypothesis for different amino-acids including methionine. A spontaneous *M*. *oryzae met1-* mutant, which is a leaky methionine auxotrophic mutant probably defective in cystathionine gamma-synthase (Cgs1, Fig 1), displayed a strong reduction in pathogenicity on rice plants [9]. In addition, a *M*. *oryzae STR3* null mutant deficient for a cystathionine beta-lyase (Cbl1, Fig 1), was also a leaky methionine auxotrophic mutant with a strong reduction in pathogenicity on rice [10]. Recently, *M*. *oryzae MET13* null mutant deficient for a methylenetetrahydrofolate reductase (Mthfr, Fig 1) was reported as a methionine auxotrophic mutant displaying a strong reduction in pathogenicity on rice [11]. The residual growth of such methionine auxotrophic mutants on a sulfate minimal medium may result from metabolic bypasses. For example, homocysteine synthase (Hcs1, Fig 1) is involved in the biosynthesis of homocysteine from sulfide and *O*-acetylhomoserine in *Aspergillus nidulans* [12]. This enzyme can bypass the metabolic defects of *met1-* and *str3-* mutants. According to these possible metabolic bypasses, we hypothesized that a methionine synthase defective mutant (Met6, Fig 1) would be a real methionine auxotrophic mutant.The first fungal methionine synthase was described in the yeast *Saccharomyces cerevisiae* Met6 [13]. Biochemical characterization of *S*. *cerevisiae* and *C*. *albicans* Met6 enzymes showed they have biochemical properties [14] similar to cobalamine-independent methionine synthases from plants [15]. This fungal enzyme (EC 2.1.1.14) catalyzes the irreversible methylation of homocysteine using a methyl derivative of tetrahydrofolate (CH3-TriGlu THF; Fig 1). Met6 is also involved into the recycling of homocysteine produced from *S*-adenosylmethionine (SAM) dependent methylation of metabolites (Fig 1).

In the present work, we identified and inactivated the *MET6* gene from *M*. *oryzae* to assay the importance of fungal methionine biosynthesis during plant infection. Expression of *MET6* was monitored during fungal growth, sporulation and appressorium formation as well as during plant infection. *MET6* deletion mutants (*Δmet6*) were obtained by targeted gene replacement. Phenotypic and metabolic analyses of these mutants showed that they were unable to synthesize methionine. These mutants were also non pathogenic on rice and barley and their pathogenicity was rescued by exogenous methionine. This result demonstrates that plant methionine sources are not available for fungal growth during infection. This study complements previous results obtained using other methionine auxotrophic *M*. *oryzae* mutants that were only partially defective in pathogenicity probably due to compensatory metabolic pathways.

## Materials and Methods

### Fungal strains, media and growth conditions

The wild type *Magnaporthe oryzae* isolate P1.2 [16] is a gift from the Centre de Coopération Internationale en Recherche Agronomique pour le Développement (CIRAD, Montpellier). P1.2 is pathogenic on rice and barley leaves and was used as a recipient strain for targeted gene replacement of *MET6*. Wild type and *Δmet6* mutants were grown at 26°C on a rice agar medium (RM) composed of rice flour (20 g.L^-1^), yeast extract (2 g.L^-1^) and agar (20 g.L^-1^) buffered to pH 5.5 with MES [2-morpholinoethanesulfonic acid] (10.66 g.L^-1^) and MOPS [3-(N-morpholino) propanesulfonic acid] (10.46 g.L^-1^). A modified Tanaka B minimal medium (MM) was used with the following composition: glucose 10 g.L^-1^ (55 mM), NaNO_3_ 2 g.L^-1^ (23 mM), KH_2_PO_4_ 2 g.L^-1^ (14 mM), MgSO_4_, 7H_2_O 0.5 g.L^-1^ (2 mM), CaCl_2_, 2H_2_O 0.1 g.L^-1^ (0.7 mM), FeSO_4_, 7H_2_O 4 mg.L^-1^ (15 μM), ZnSO_4_, 7H_2_O 8 mg.L^-1^ (55 μM), CuSO4, 5H_2_O 0.6 mg.L^-1^ (2.5 μM), H_3_BO_3_ 0.1 mg.L^-1^ (1.4 μM), MnSO_4_, H_2_O 0.2 mg.L^-1^ (1.3 μM), NaMoO_4_, 2H_2_O 0.15 mg.L^-1^ (0.5 μM), thiamine (1 mg.L^-1^), biotin (5 μg.L^-1^) and agarose (8 g.L^-1^). The complete medium (CM) was composed of the above MM supplemented with yeast extract (2 g.L^-1^). The MM was also supplemented with methionine (Sigma, France), cysteine (Merck, France), homocysteine (Fluka, Switzerland), glutathione (Sigma, France), betaine (Sigma, France), *S*-adenosylmethionine (Boeringher, France), or *S-*methylmethionine (Boeringher, Ingelheim France) at a concentration of 1 mM unless otherwise specified. *M*. *oryzae* liquid cultures were grown in 6-well tissue culture plates (Greiner Bio One, Germany) containing 4 mL of liquid medium. Each well was inoculated with 10 calibrated mycelial plugs from a 7 day old CM agar culture. Sporulation was obtained on RM medium supplemented with 1 mM methionine 10–15 days after inoculation. Transformants were stored as dried mycelia on filter paper disks at -20°C [16]. *Escherichia coli* strain DH5α was used as host for cloning and plasmid propagation and was grown at 37°C in Luria-Bertani medium with appropriate antibiotics.

### Pathogenicity assays

Pathogenicity assays were performed using the susceptible barley (*Hordeum vulgare L*.) cultivar Express and the susceptible rice (*Oryza sativa*) cultivar Sariceltik. Barley seedlings were grown at 15°C with 60% humidity for 2 to 3 weeks. Rice seedlings were cultivated for 20 to 30 days at 70% relative humidity and 25°C day/20°C night in a phytotronic growth chamber. Fungal spores were harvested from 10- to 14-day old *M*. *oryzae* grown on RM medium supplemented with 1 mM methionine. Leaf segments (3 cm long) from barley (2 leaves stage) were placed on water agar (1% w/v) containing kinetin (2 mg.L^-1^), inoculated with 35 μL droplets of a *M*. *oryzae* spore suspension (3.10^4^ spores.mL^-1^) and incubated at 26°C under a photoperiod of 12 h light. Leaf symptoms were recorded from 3 to 7 days after inoculation. Penetration rate of *M*. *oryzae* into barley epidermal cells or onion epidermis were assessed 24 hours after drop inoculation on detached leaves using a bright field Zeiss microscope, as described [16]. Spray inoculation of barley (2 leaves stage) and rice leaves (3/4 leaves stage) was performed with a spore suspension (3.10^5^ spores.mL^-1^) containing 0.5% gelatin (w/v). A total volume of 10 mL of spore suspension was used for each pot containing five plants. Inoculated barley or rice plants were first transferred to a humid chamber with 100% humidity at 20–22°C in darkness for 24 hours and then to a greenhouse at 23°C. Leaf symptoms were recorded 5–10 days after inoculation.

### Nucleic acid manipulations, PCR amplification and Southern blots

Plasmid DNAs were recovered from bacterial cultures using the QIAprep Spin Miniprep kit (Qiagen France) following the manufacturer’s instructions. Genomic DNA was isolated from *M*. *oryzae* mycelium as described [17], with the following modifications. Mycelium from 3-day old CM liquid cultures were lyophilized and ground to a fine powder using a bead grinder [18]. DNA was extracted at 65°C for 30 min with a buffer composed of Tris-HCl 100 mM pH 7.5, EDTA-Na_2_ 50 mM, SDS 1% (w/v) and NaCl 500 mM. PCR amplifications were performed using a PTC-100 (MJ Research, USA) with the primers listed in S1 Table. The *Pfu* Turbo High fidelity DNA polymerase (Stratagene, USA) was used with the following program: 2 min at 94°C, followed by 30 cycles of 30 s at 94°C, 30 s at 60°C, 1 min at 72°C and a final step at 72°C. Other DNA amplifications were performed with *Taq* polymerase (Invitrogen, France). Sequencing reactions were performed by Genome Express S.A. (Meylan, France). Sequences were analyzed with Vector NTi Suite9 (Invitrogen, France). For Southern hybridization, *M*. *oryzae* genomic DNA fragments digested with *Bam*HI were separated by electrophoresis on 1% agarose gel and transferred to a Nylon membrane (Hybond N^+^, Amersham Bioscience, U.S.A.) with a vacuum blotter (Appligen, Belgium). The right border of *MET6* CDS was obtained by PCR amplification using MET6-3 and MET6-4 primers (S1A Table, S1 Fig) and *M*. *oryzae* genomic DNA as template. This fragment was labeled with ^32^P using the Megaprime DNA Labeling kit (Amersham Bioscience, U.S.A.) and used as a probe for Southern hybridization with standard procedures [19].

### Construction of *M*. *oryzae MET6* deletion mutants of and their complementation

The *MET6* gene replacement construct was generated as follows. A 1.4-kb fragment (LB-MET6) corresponding to a region upstream of the *MET6* start codon was amplified from P1.2 genomic DNA using primers MET6-1 and MET6-2 (S1A Table, S1 Fig). A 1.2-kb fragment (RB-MET6) downstream of the *MET6* stop codon was amplified using primers MET6-3 and MET6-4 (S1A Table, S1 Fig). The 1.4-kb *hph* cassette was amplified from plasmid pCB1003 [20]. The 1.4-kb LB-MET6 fragment was digested with the restriction enzymes *Eco*RI / *Sac*II while the amplified *hph* cassette was digested with *Sac*II / *Bgl*II. Both DNA fragments were ligated into the pDHT-Kan vector (SK581, gift of S. Kang, University of Pennsylvania, USA) digested with *Bam*HI and *Eco*RI to give an intermediary vector containing the 1.4-kb LB-MET6 fused to the *hph* cassette. The blunt 1.2-kb RB-MET6 PCR fragment was ligated to the previous plasmid digested with *Pme*I to give the *MET6* gene replacement plasmid pMET6-delta. A final PCR amplification was performed using primers MET6-5 and MET6-6 (S1A Table, S1 Fig) and pMET6-delta as template to yield a linear 4.4-kb *MET6* gene replacement construct.

Preparation and transformation of *M*. *oryzae* protoplasts were performed as described [16]. Hygromycin-resistant colonies were transferred to CM and MM containing hygromycin (120 mg.L^-1^) to select stable transformants. Transformants were purified by isolation of single spore germinating on water agar (3 g.L^-1^) supplemented with 1 mM methionine under a binocular. To test the integration of HYG (hph cassette) at the *MET6* locus, PCR was performed using respectively primers MET6.10 / HYG(-) and HYG(+) / MET6.7. For complementation, a 6.0-kb PCR product containing the *M*. *oryzae* wild type *MET6* gene (1.95 kb upstream start codon, 2.4-kb CDS and 1.75 kb downstream of stop codon) was amplified from P1.2 genomic DNA using primers MET6-7 and MET6-10 (S1A Table, S1 Fig). This PCR fragment was cloned in a pTOPO blunt vector (Invitrogen) to give pTOPO-MET6 that was further digested with *Cla*I and *Bam*HI. The resulting 5.5-kb *Cla*I-*Bam*HI fragment was cloned into the pCB1635 vector that contains a *BAR* gene conferring bialaphos resistance [20] to give pCB1635-MET6. This complementation vector was linearized by *Not*I digestion and introduced by protoplast transformation into the 15.1 *Δmet6* mutant. Transformants were selected using the BAR selective medium [20] containing 35 mg.L^-1^ bialaphos. The presence or absence of the *MET6* gene in *Δmet6* P1.2 mutants 4.1, 15.1, 22.1 and 23.1, ectopic P1.2 transformants, wild type P1.2 and *Δmet6* transformants 15.1 complemented with pCB1635-MET6 was verified by PCR amplification using primers MET6.8 and MET6.9 (S1A Table) and by Southern hybridization (S2 Fig).

### Expression profiling by real time RT-PCR

Total RNA was extracted from *in vitro* grown mycelium, ungerminated spores, young appressoria (12 hours) and mature appressoria (24 hours) and from infected leaves of the susceptible rice cultivar Sariceltik (3, 6 and 8 days after infection), using different protocols as described below. The mycelium was grown as a stationary culture in different liquid media for 6 days and total RNA was extracted from lyophilized mycelium using the Qiagen RNeasy Plant mini kit following manufacturer’s instructions. Spores were harvested from 7–9 days old RM cultures supplemented with methionine, resuspended in water containing 10 μM of 1,16-hexadecanediol at a concentration of 1.10^5^ spores.mL^-1^ and placed as 35 μl drops on a Teflon membrane. These membranes were incubated at 26°C for 12 or 24 hours to obtain young and mature appressoria, respectively, as described [16]. RNA from spores and appressoria was extracted using the hot acid phenol protocol [16]. RNA from infected rice leaves was extracted by the phenol-LiCl method [16]. Reverse transcriptase-polymerase chain reaction (RT-PCR) was performed using 5 μg of total RNA as starting material and the Thermoscript RT-PCR system kit (Invitrogen, France) according to the manufacturer’s instructions. Real time PCR was performed in 96-well plates using an ABI-7900 (Applied Biosystem, France), with water as negative control and cDNAs from mycelium grown on complete medium as positive control. Primer pairs were designed using Primer Express Software (Applied Biosystem, France). Primer efficiency was determined with different concentrations of forward and reverse primers (50 to 900 nM) using genomic DNA or mycelium derived cDNA as templates. A 10^–3^ cDNA dilution was used for fungal tissues (mycelium, spores, appressorium) while a 10^–2^ cDNA dilution was used for infected leaves. Standard real time PCR conditions were used with SYBR green PCR Master Mix (Applied Biosystem, France) with the following program: 95°C for 10 min and 40 cycles of 95°C for 30 s, 60°C for 1 min and 72°C for 30 s. Ct computations were carried out as previously described [21]. Internal *M*. *oryzae* controls corresponding to constitutively expressed *M*. *oryzae* genes encoding either actin (*ACT1*, MGG_03982.7) or ketol-acid reductoisomerase (*ILV5*, MGG_15774.7), were used as described [21]. qPCR primers are listed in S1B Table.

### Extraction and quantification of sulfur-containing metabolites

Mycelium was grown in liquid MM supplemented with 1 mM methionine or additional metabolites as specified. The mycelium was collected after 8 to 10 days of growth until no methionine was detected in the culture medium. Mycelial mats were washed with distilled water and lyophilized. Dried mycelia were grinded into a fine powder using a bead grinder [18]. Extraction of metabolites was performed on ice with a solution composed of 50 mM HCl and 50% acetonitrile (ratio of 20 mg of dry tissue per mL of solution). The final suspension was clarified by centrifugation (16,000 *g*) for 10 min and directly used for quantification of sulfur-containing metabolites using HPLC. Separation of methionine and cystathionine was achieved as described [22]. Fluorescence of the OPA-adducts was monitored at 455 nm upon excitation at 340 nm using a SFM25 fluorimeter (UVK-Lab, France). Thiol-containing molecules were converted into their bimane derivatives using monobrobimane (mBBr, thiolyte, Calbiochem, USA) as described [23]. Bimane derivatives were separated using the following elution protocol with solvents A and B composed of 0.1% (v/v) trifluoroacetic-water and HPLC-grade methanol, respectively. The Uptisphere UP5HDO-25E column (Interchim, France) was operated at 25°C with a flow rate of 1 mL.min^-1^ and with the following gradient: 0 min, 18% B; 7 min, 18% B; 37 min, 20% B; 47 min, 80% B; 48 min, 100% B; 53 min, 100% B; 54 min, 18% B; 60 min before a new injection. *S*-adenosylmethionine and *S*-adenosylhmocysteine derivation was achieved by incubation of 0.050 ml of acetonitrile-HCl extract in presence of 0.1 ml of 0.2 M perchloric acid, 0.0125 ml of 3 M sodium acetate and 0.025 ml of 1.5 M chloro-acetaldehyde solution for 4 h at 40°C [24]. Fluorescent 1,N^6^-etheno derivatives were separated using a Nucleodur Pyramid C18 (4 x 250 mm) column (Macherey-Nagel, France) using the following elution protocol with solvent A and B composed of 50 mM NaH_2_PO_4,_ pH 4.5 and 100% acetonitrile respectively. The elution was operated at 25°C with a flow rate of 1 mL.min^-1^ and with the following gradient: 1 min 3% B; 12 min, 20% B; 14 min, 70% B; 18 min, 70% B and then 18 min, 3% B, 30 min before a new injection. Fluorescence of the isoindoles was monitored at 410 nm upon excitation at 270 nm using a SFM25 fluorimeter (UVK-Lab, France). Identity of these compounds was assessed by co-injection of authentic standards. Quantification was performed by measuring peak areas with 450-MT2 software (UVK-Lab, France) and expressed as nmoles/mg dry weight.

### Bioinformatics

Available fungal genome sequences were searched using different Blast algorithms at the NCBI (http://www.ncbi.nlm.nih.gov/BLAST), Broad Institute (http://www.broad.mit.edu/annotation/fungi) and JGI (http://genome.jgi-psf.org/euk_home.html) websites. The *M*. *oryzae* genome sequence version 8 and the corresponding CDS and gene nomenclature (v7) available at the Broad Institute, were used in this study. *M*. *oryzae* ESTs were searched using BlastN at the NCBI and COGEME (http://cogeme.ex.ac.uk) websites. Protein sequence alignments and phylogenetic trees were obtained using ClustalW and MEGA 5.1 [25].

## Results

### Characterization of the *M*. *oryzae MET6* gene encoding a cobalamine-independent methionine synthase

**Fig 2:**
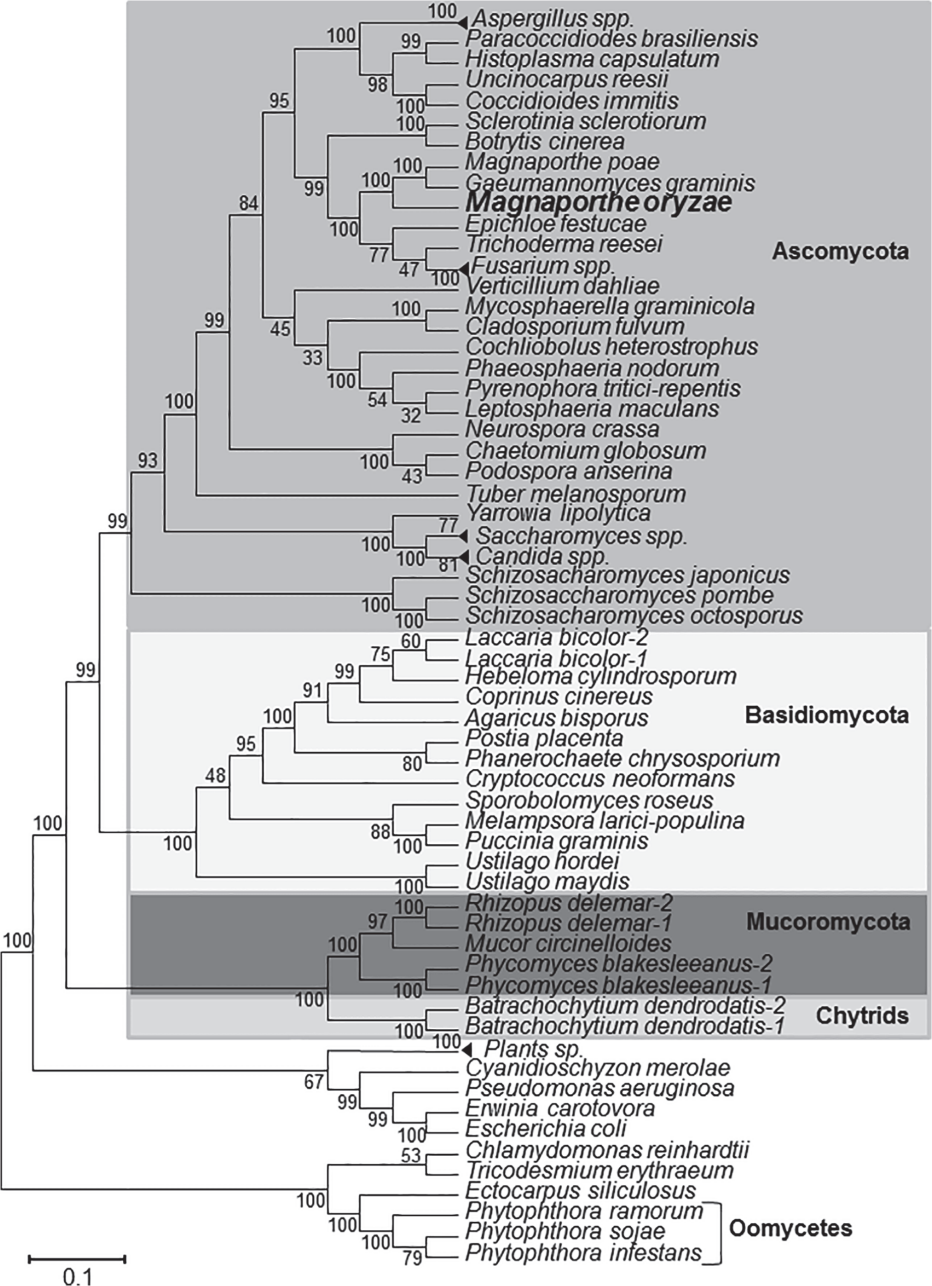
Phylogenetic tree of fungal cobalamine-independent methionine synthases. Fungal cobalamine-independent methionine synthases were searched in available fungal protein databases using BlastP and *Aspergillus nidulans* METH (AAF82115) or *Saccharomyces cerevisiae* Met6p (YER091c) protein sequences as queries. Alignment was performed using Clustal W and blosum 62 matrix. The phylogenetic tree was constructed using the Minimum Evolution Method with pair wise deletion for gaps, missing data Poisson correction, and 2000 replicates for bootstraping. Accession numbers are listed in Table 2.

The protein sequence of *Aspergillus nidulans* methionine synthase METH [26] was used as query for BlastP searches of the *M*. *oryzae* protein database at the Broad Institute. A single protein sequence encoded by the gene MGG_06712.7 was identified with 80% amino acid identity and 87% similarity to *A*. *nidulans* METH protein sequence. This gene was named *MET6* following the nomenclature used for the *S*. *cerevisiae* cobalamine-independent methionine synthase [13, 26]. Aligment of *M*. *oryzae* Met6 protein sequence with *N*. *crassa* (AF4040820_1), *A*. *nidulans* (AF275676_1), *S*. *cerevisiae* (YER091c) and cytoplasmic *A*. *thaliana* Met6 sequences (S3 Fig) revealed identities ranging respectively from 76, 80, 58 and 48%. The predicted structure of *MET6* including start codon, stop codon, intron positions and splicing sites was confirmed by alignment of its genomic sequence to ESTs from public databases. This comparison highlighted a partial 78-bp 5’UTR and a full length 531-bp 3’UTR (S1 Fig). Met6 protein sequence Interpro analysis identified a C-terminal domain (PFAM PF01717) and an N-terminal domain (PFAM PF08267) typical of methyltransferases. These two domains are characteristic of cobalamine-independent methionine synthases. In particular, these C-terminal domains contain conserved amino-acids involved in zinc binding, interaction with homocysteine and methyl-triglutamate-tetrahydrofolate [27]. Met6 is likely a cytoplasmic enzyme since it has no signal peptide at its N-terminus region. Using BlastP search, we identified a single gene encoding a cobalamine-independent methionine synthase in almost all fungal genomes. Phylogenetic analysis shows that *M*. *oryzae MET6* belongs to a family of conserved orthologous genes (Fig 2). In few fungal species such as *Laccaria bicolor*, *Rhizopus oryzae*, *Phycomyces blackesleeanus* and *Batrachochytrium dendrobatidis*, we identified two *MET6* paralogues. We have not identified cobalamine-dependent methionine synthase encoding genes in *M*. *oryzae*, nor in other fungi.

**Fig 3:**
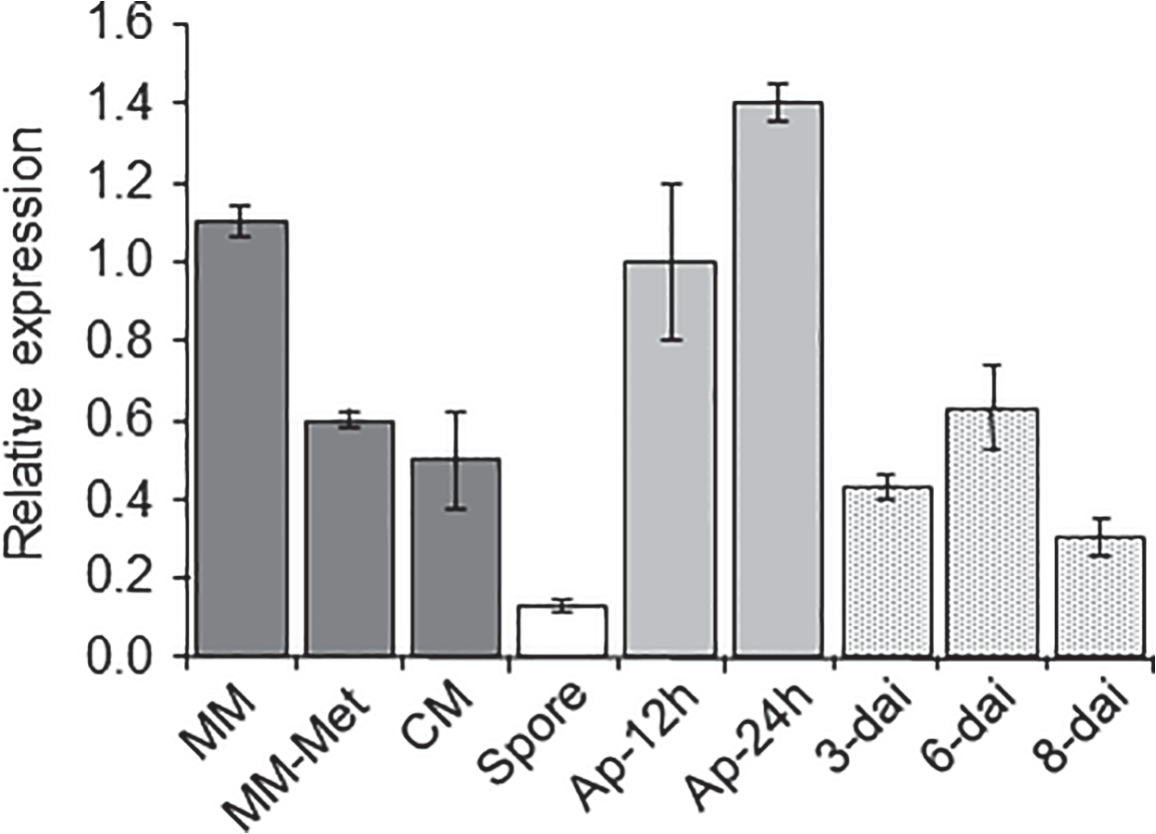
Expression of *MET6* during *M*. *oryzae* development and rice infection. Transcripts from *M*. *oryzae MET6* were monitored by qRT-PCR. *MET6* was expressed at high levels in mycelium grown on MM and in appressoria, and was down-regulated in mycelium grown on MM + Met and CM (2-fold) and during infection (2-fold). A strong down-regulation was observed in dormant spores (10-fold). RNA was extracted from wild type P1.2: mycelium grown on minimal medium (MM), minimal medium supplemented with 1 mM methionine (MM-Met), complete medium (CM), spores (Spore), 12-hours (young) and 24-hours (mature) old appressoria differentiated on Teflon (Ap-12h and Ap-24h) and infected leaves 3-, 6-, and 8-days after inoculation (dai) of the susceptible rice cultivar Sariceltik (spray inoculation). Primers are listed in Table 1B. Gene expression was normalized relative to the level of the constitutive gene *ILV5* (MGG_15774.7). Each bar is the mean value of three replicates with standard deviations as vertical lines.

Expression of *MET6* was monitored during *M*. *oryzae* development and plant infection using real time RT-PCR (Fig 3). During fungal growth in MM liquid culture, *MET6* was expressed to a similar level as the reference gene *ILV5* [21]. Addition of 1 mM methionine (MM + Met) or yeast extract (CM contains up to 300 μM methionine) reduced *MET6* expression by 2-fold (Fig 3). *MET6* has its lowest level of expression in spores (10-fold lower than MM mycelium). In appressoria, expression of *MET6* was similar to MM mycelium. During infection, *MET6* was expressed at levels similar to mycelium grown on CM. Overall, *MET6* was expressed at high levels in mycelium grown on MM and in appressoria, and displayed a slight down regulation in mycelium grown on methionine rich media (MM + Met, CM: 2-fold) and during infection (2-fold). A strong down-regulation was observed in dormant spores (10-fold compared to mycelium). This down regulation in spores was not observed for the qRT-PCR reference gene *ILV5* involved the biosynthesis of hydrophobic amino-acids.

**Table 1:** Nutrient requirements of *M*. *oryzae Δmet6*mutants.

Growth medium	Wild type	*Δmet6*
Minimal medium (MM)	**+++**	**-**
MM + Cysteine, 1mM	**+++**	**-**
MM + Glutathione, 1mM	**+++**	**-**
MM + Cystathionine, 1 mM	**+++**	**-**
MM + Homocysteine, 1 mM	**-**	**-**
MM + Homocysteine, 0.1 mM	**++**	**-**
MM + Methionine, 1 mM	**+++**	**+++**
MM + *S*-adenosylmethionine (SAM), 1 mM	**+++**	**+**
MM + *S-*methylmethionine (SMM), 1 mM	**+++**	**+**
MM + Methylthioadenosine (MTA), 1 mM	**+++**	**+**
MM + Betaine, 1 mM	**+++**	**-**
MM + Choline, 1 mM	**+++**	**-**

Wild type (P1.2) and *Δmet6 M*. *oryzae* mutants (M15.1, M22.1, M23.1) were grown on minimal medium (MM) supplemented with different sulfur metabolites. Mycelium growth was observed 4 to 6 days after transfert of a mycelial plug from CM to MM. MTA was dissolved in dimethylsulfoxide (DMSO) and added to MM at 1% DMSO final concentration.

+++, normal growth

++, reduced growth

+, highly reduced growth

-, no growth.

### Construction and phenotypic characterization of *M*. *oryzae MET6* deletion mutants

**Fig 4:**
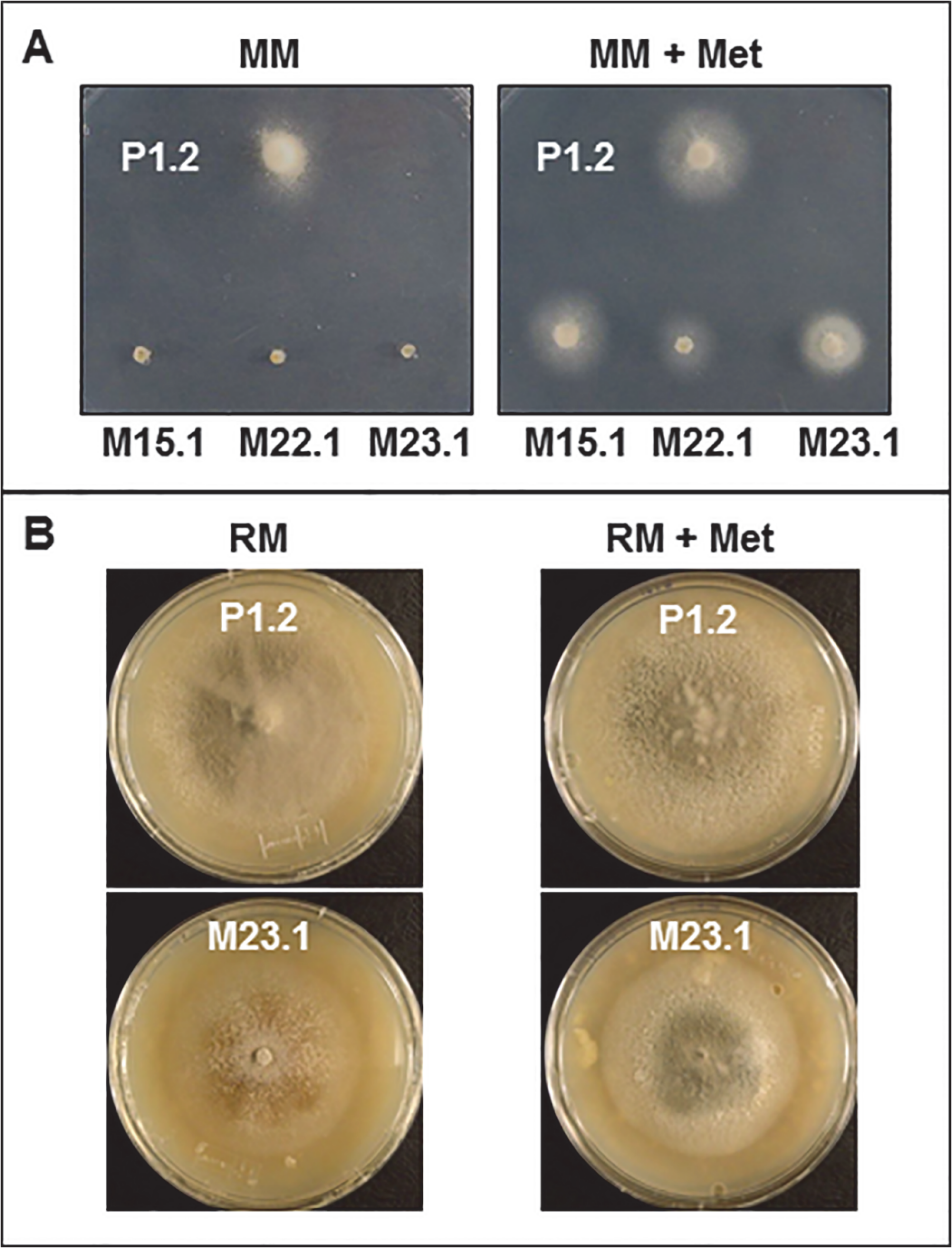
Phenotypes of *M*. *oryzae Δmet6* mutants in axenic cultures. **A**, *M*. *oryzae* wild type (P1.2) and *Δmet6* mutants (M15.1; M22.1 and M23.1) were grown on minimal medium (MM) with sulfate as sole sulfur source (left) or in MM supplemented with 1 mM methionine (MM + Met, right). *Δmet6* mutants were unable to grow on MM, but grew normally on MM + Met. **B**, *M*. *oryzae* wild type (P1.2) and *Δmet6* mutant (M23.1) were grown on rice agar medium (RM) in the absence or presence of 1 mM Met (RM + Met). *Δmet6* mutant M23.1 displayed a radial growth similar to WT on RM, but developped a brown-orange color differing from the grey color of WT. Addition of methionine to MM rescued the color phenotype of *Δmet6*.

*MET6* null mutants were obtained by targeted gene replacement of *MET6* by a hygromycine resistance gene (S1 Fig) in wild type *M*. *oryzae* strain P1.2 pathogenic on rice and barley [16]. Hygromycin-resistant transformants were transferred to minimal media supplemented or not with methionine. 20% of transformants were unable to grow on MM, but displayed a normal growth on MM supplemented with methionine, as expected for methionine auxotrophic mutants. PCR analysis of *MET6* loci in four transformants auxotrophic for methionine (M4.1, M15.1, M22.1 and M23.1) showed that *MET6* open reading frame was replaced by the hygromycin resistance gene. Southern blot analysis of these transformants confirmed gene replacement at *MET6* locus, as fragments of expected size were observed in *Δmet6* transformants compared to wild type and ectopic transformant E19.1 (S2 Fig). The additional band observed for ectopic transformant E19.1 corresponds to the integration of the gene replacement vector at another locus than *MET6* (S2 Fig). These methionine auxotrophic transformants unable to grow on MM, were rescued by at least 0.1 mM exogenous methionine (Fig 4A). For all experiments, we added 1 mM methionine to media used for growing methionine auxotrophic transformants. One of these auxotrophic mutants (*Δmet6-15*.*1*) was complemented with the pCB1635*-*MET6 vector conferring bialaphos resistance and carrying a wild type *MET6* allele. Fifty percent of the bialaphos resistant transformants (*Δmet6-15*.*1*:*MET6*) displayed the same growth rate as wild type strain P1.2 on MM showing that the *MET6* wild type allele complemented the methionine auxotrophy of *Δmet6-15*.*1* (data not shown). Precursors of methionine such as cysteine, cystathionine and homocysteine, as well as sulfur-containing metabolites such as glutathione, choline and betaine [29], were added to MM at 1 mM. These compounds did not restore the growth of *Δmet6* mutants on MM (Table 1). Since homocysteine was reported to be toxic to fungi [29, 30], we tested its effect on wild type growth. 1 mM homocysteine inhibited growth of *M*. *oryzae* on MM, while 0.1 mM only partially inhibits growth (Table 1 and S4 Fig). At this sub toxic concentration, homocysteine did not recue *Δmet6* mutants on MM (Table 1, S4 Fig). Addition of methionine derivatives such as SAM (*S*-adenosylmethionine, [31].), SMM (*S*-methylmethionine, [32]) and MTA (methylthioadenosine) to MM at 1 mM very partially supported the growth of *Δmet6* mutants (Table 1 and S6 Fig). These experiments showed that growth of *Δmet6* mutants on MM was rescued by methionine and partially by its derivatives (SAM, SMM and MTA).

The *Δmet6* mutants displayed additional developmental phenotypes. On RM, they differed from wild type in their morphology, although their radial growth was similar (Fig 4B). Mutants had only few aerial hyphae, were less melanized, accumulated brown-orange pigments and had a very low sporulation rate (100-fold lower than wild type, data not shown). The pigmentation and sporulation defects of *Δmet6* mutants was almost restored (3-fold lower than wild type) by the addition 1 mM of methionine to rice agar medium (RM + Met, Fig 4B). The complemented *Δmet6-15*.*1* mutant (*Δmet6*-*15*.*1*:*MET6*) displayed the same mycelial growth, morphology, pigmentation and sporulation rates as wild type, demonstrating that the pleiotropic phenotype of *Δmet6* mutants clearly results from the inactivation of *MET6* (S5 Fig).

### Pathogenicity defects of *MET6* deletion mutants on rice and barley

**Fig 5:**
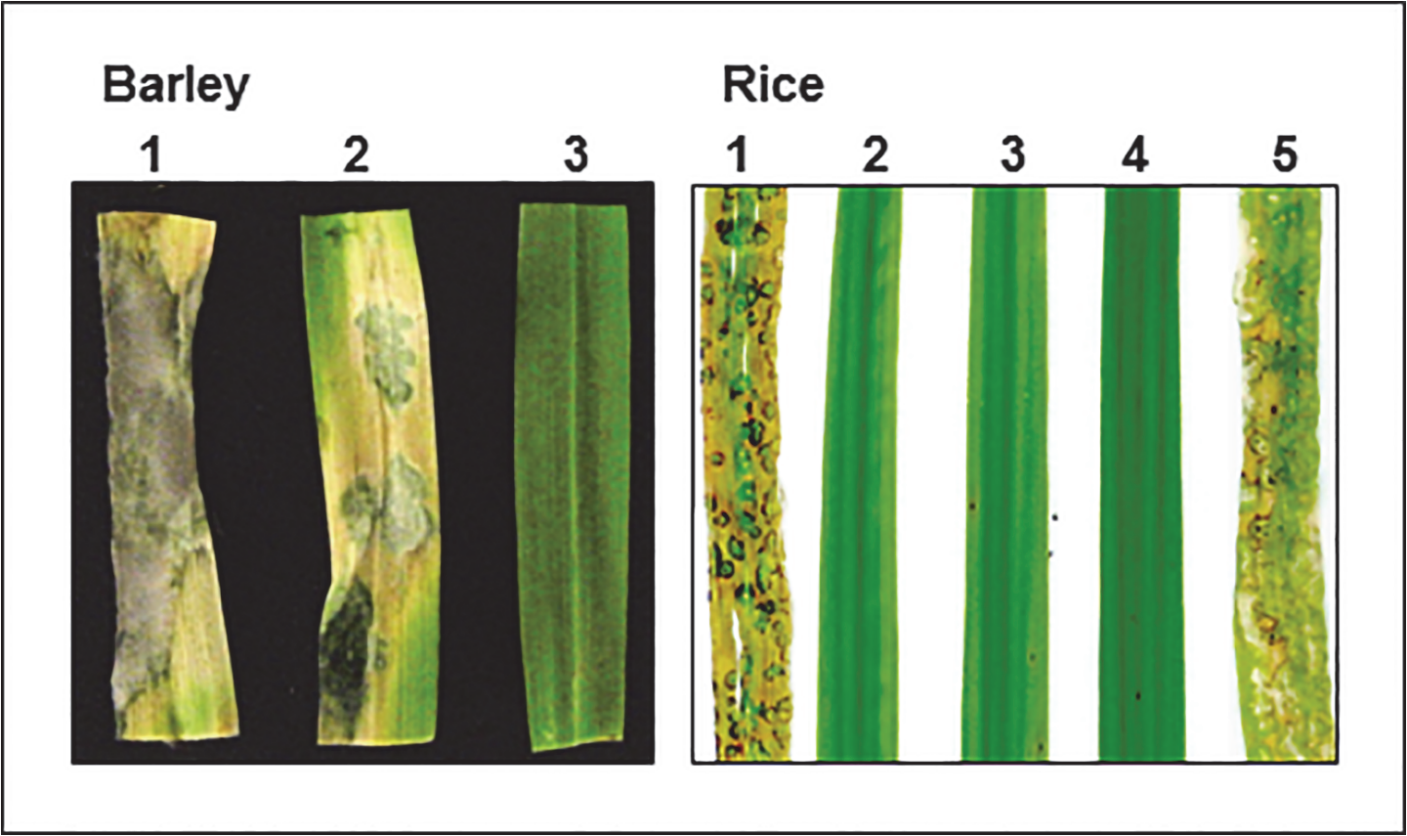
Pathogenicity of *M*. *oryzae Δmet6* mutants on barley and rice. Left, Detached Barley leaves (cv. Express) were inoculated with droplets of spore suspension of *M*. *oryzae* wild type P1.2 (1) and *Δmet6* mutant M23.1 in the presence (2) or absence (3) of 1 mM methionine. *Δmet6* mutant M23.1 was non-pathogenic on detached barley leaves (no lesion). Addition of 1 mM methionine to *Δmet6* spores rescued its pathogenicity defect. Right, Young rice plants (cv. Sariceltik, 4 leaves stage) were spray inoculated with spore suspensions of wild type P1.2 (1), three *Δmet6* mutants (2: M15.1; 3: M22.1; 4: M23.1) and a transformant corresponding to the complementation of *Δmet6* mutant (M15.1) with *MET6* wild type allele (5). *Δmet6* mutants M15.1, M22.1 and M23.1 were non-pathogenic on rice leaves (no lesion). Complementation of *Δmet6* mutants M15.1 with wild type *MET6* allele rescued its pathogenicity defect.

*Δmet6* mutants M15.1, M22.1 and M23.1 were non-pathogenic on detached barley leaves (Fig 5) even with a high inoculum (3.10^5^ spores.mL^-1^). Addition of 1 mM methionine to *Δmet6* spores rescued the pathogenicity defect of these mutants that developed typical susceptible blast lesions on barley leaves (Fig 5). However, these lesions differed from those of wild type, as they did not support sporulation. Susceptible lesions were also induced after inoculating *Δmet6* mutants with 1 mM SMM, while no symptoms were obtained by adding SAM and MTA (S6 Fig). Spray-inoculation of spores from *Δmet6* mutants onto leaves of the susceptible rice cultivar Sariceltik did not induce any lesions demonstrating that these mutants were also unable to infect rice leaves (Fig 5). The *Δmet6*-*15*.*1*:*MET6* complemented transformants were as pathogenic as wild type (Fig 5), confirming that pathogenicity defects of *Δmet6* mutants results from *MET6* inactivation.

The ability of *Δmet6* mutants to differentiate appressoria was assessed using spore suspensions deposited on Teflon membranes (Table 2). Spore germination of *Δmet6* mutants was slightly reduced (1.5-fold) compared to wild type, while the percentage of appressorium formation was not signicantly affected. On barley leaves and onion epidermis, *Δmet6* mutants differentiated appressoria that were unable to penetrate into plant epidermal cells, even 48 hours after inoculation (hai), although a large number of appressoria were differentiated on the leaf surface (Table 2, S7 Fig). In the same assay, more than 90% of wild type appressoria allowed the penetration of the fungus into barley leaves at 24 hai. These results show that *Δmet6* appressoria were not functional. Wounding of barley leaves did not rescue the pathogenicity defect of *Δmet6* mutants, suggesting that these mutants are also defective in invasive infectious growth. Addition of methionine to *Δmet6* spore suspension restored its pathogenicity on wounded leaves (data not shown). These results demonstrate that *Δmet6* mutants are unable to invade either intact or wounded plant leaves, unless fed with exogenous methionine.

**Table 2:** Appressorium differentiation and penetration in *M*. *oryzae Δmet6* mutants.

	Teflon	Teflon	Barley leaves
Strains	Spore germination, %	Appressorium differentiation, %	Successful penetration, %
Wild type P1.2	80	80	100
*Δmet6* M15.1	70	80	na*
*Δmet6* M22.1	62	63	na*
*Δmet6* M23.1	37	80	0
Ectopic E19.1	80	80	100

Spores of wild type P1.2, *Δmet6* mutants (M15.1, M22.1, M23.1) and ectopic P1.2 transformant E19.1 were deposited on a Teflon membrane with hexadecanediol or on detached barley leaves kept on kinetin water agar plates. Spore germination and appressorium formation were observed using a bright field microscope. Epidermal layer was stripped 24–30 h after inoculation. Appressoria formed on barley leaves were stained with Cotton blue in lactic acid and observed using a bright field microscope. Penetration was scored as successful if unstained infectious hyphae were detected inside epidermal cells underneath appressoria (Fig 7). *Δmet6* mutants differentiated appressoria at normal rate that were unable to penetrate into plant epidermal cells. na*: not analyzed

### Metabolic and gene expression signatures of *MET6* deletion mutants

Sulfur metabolites were quantified in 8 day-old mycelia of wild type *M*. *oryzae* grown in still liquid MM with or without 1 mM methionine. This stage corresponds to the end of the exponential phase (reduced growth), and no methionine was detectable in culture media by HPLC, indicating that it has been used by fungal cells. Wild type mycelia grown on MM + Met accumulated larger amounts of cystathionine (CTT, 3-fold) and homocysteine (Hcys, 1.5-fold) than on MM, while glutathione (GSH), cysteine (Cys) and methionine (Met) levels were unchanged (Table 3). In addition, *S*-adenosylmethionine (SAM) levels were slightly increased (1.3 fold) in wild type grown on MM + Met compared to MM (Table 4). When grown on MM supplemented with 1 mM methionine, *Δmet6* mutants accumulated large amounts of homocysteine and cystathionine (20- and 10-fold respectively), and to a lesser extent cysteine (2.6-fold), compared to wild type (Table 3). However, methionine and GSH levels were similar to wild type (Table 3). In addition, *S*-adenosylmethionine (SAM) and *S*-adenosylhomocysteine (SAH) levels increased respectively by 3 and 5-fold in *Δmet6* mutants compared to wild type (Table 4). This result suggests that exogenous methionine is transformed into SAM and SAH that accumulate as a consequence of the absence of Met6. However, the methyl index (SAM/SAH) of *Δmet6* remains close (2.2 to 3.6) to wild type index (4.85; Table 4).

**Table 3:** Effects of exogenous methionine on sulfur amino acids and glutathione levels in *M*. *oryzae Δmet6* mutants and wild type.

*nmoles.mg^-1^ dry weight	GSH	Cys	CTT	Hcys	Met
Wild type P1.2, MM	*11 ± 2	0.2 ± 0.08	1.5 ± 0.4	0.06 ± 0.04	1.5 ± 0.4
Wild type P1.2, MM + Met	9.3 ± 2	0.1 ± 0.06	4.2 ± 1.4	0.1 ± 0.05	1.3 ± 0.4
**Fold change** ** P12-Met/P12	**0.85**	**0.5**	**2.8** °	**1.6** °	**0.85**
*Δmet6* M15.1, MM + Met	9.5 ± 2	0.3 ± 0.16	41 ± 6.7	1.9 ± 0.4	1.5 ± 0.7
*Δmet6* M22.1, MM + Met	8.9 ± 2	0.3 ± 0.15	38.5 ± 3	1.9 ± 0.8	1.0 ± 0.3
*Δmet6* M23.1, MM + Met	8.7 ± 2	0.2 ± 0.02	38 ± 4.0	2.2 ± 0.7	1.2 ± 0.2
**Fold change** ** Δ*met6* / P1.2	**0.95**	**2.6** °	**9.3** °	**20** °	**0.95**

Wild type *M*. *oryzae* isolate P1.2 was grown in liquid medium in absence (MM) or presence of 1 mM methionine (MM + Met) for 8 days. The *Δmet6* mutants (M15.1, M22.1 and M23.1) were grown in liquid MM supplemented with 1 mM methionine (MM + Met) for 8 days. Cys (cysteine), CTT (cystathionine) and Hcys (homocysteine) were quantified by HLPC as described in Materials and Methods. Data were expressed in nmoles.mg^-1^ dry weight and represent the mean (± standard deviation) of two technical replicates derived from three biological replicates.

**Fold change **(**Δ*met6* / P1.2 MM + Met) was calculated using average value of the three mutant lines over wild type.

°Differences are significant according to a t-test (5%).

**Table 4:** Effects of exogenous methionine on methyl cycle metabolites levels in *M*. *oryzae Δmet6* mutants and wild type.

*nmoles.mg^-1^ dry weight	SAM	SAH	SAM/SAH
Wild type P1.2, MM	*0.27 ± 0.1	0.07 ± 0.02	3.85
Wild type P1.2, MM + Met	0.35 ± 0.1	0.07 ± 0.02	4.85
**Fold changes** ** P12 + Met / P12	**1.3** °	**1**	**1.26**
*Δmet6* M15.1, MM + Met	1.25 ± 0.3	0.45 ± 0.16	2.7
*Δmet6* M22.1, MM + Met	1.10 ± 0.17	0.30 ± 0.05	3.6
*Δmet6* M23.1, MM + Met	0.75 ± 0.1	0.35 ± 0.06	2.2
**Fold changes** ** Δ*met6* / P1.2	**3.0** °	**5.3** °	**0.56**

Wild type *M*. *oryzae* isolate P1.2 was grown in liquid medium in absence (MM) or presence of 1 mM methionine (MM + Met) for 8 days. *Δmet6* mutants (M15.1, M22.1 and M23.1) were grown in liquid MM supplemented with 1 mM methionine (MM + Met) for 8 days. SAM and SAH were quantified by HPLC as described in Materials and Methods. Data were expressed in nmoles.mg^-1^ dry weight and represent the mean ± standard deviation of 8 technical assays derived from three biological replicates.

Fold changes** (Δ*met6* / P1.2 MM + Met) was calculated using average value of the three mutant lines over wild type.

°Differences are significant according to a t-test (5%).

**Fig 6:**
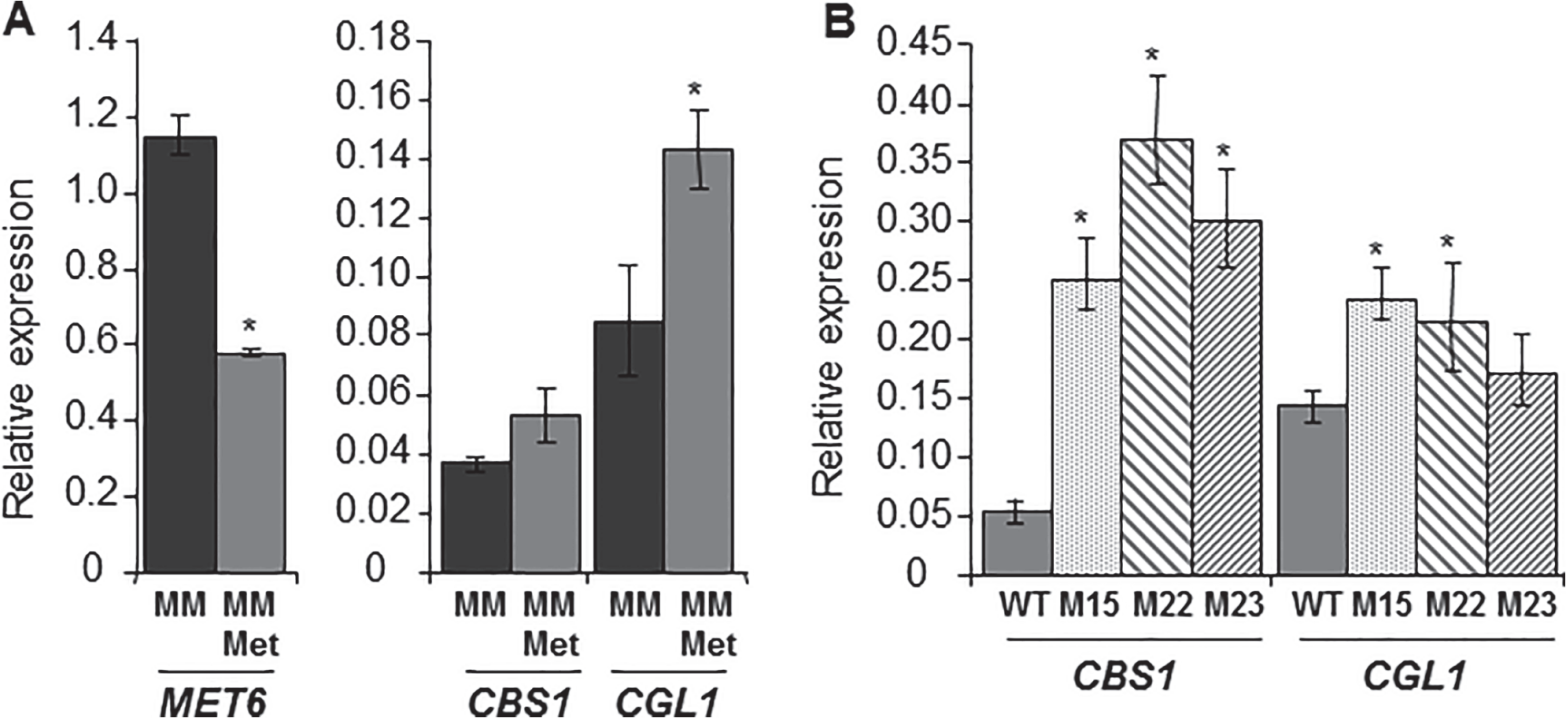
Expression profiles of *MET6* and genes from reversed transulfuration pathway in *M*. *oryzae Δmet6* mutants and wild type. **A,** Transcript levels in mycelium of wild type P1.2 isolate grown in MM (MM: black bar) or in MM supplemented with 1 mM methionine (MM-Met: grey bar). Addition of methionine to MM down-regulated *MET6* (2 fold) and up-regulated *CGL1* fro reverse transsulfuration pathway (1.7 fold). **B,** Transcript levels in mycelium of wild type P1.2 (grey bar) and three *Δmet6* mutants (M15.1: punctuated grey bar; M22.1: left shaded grey bar; and M23.1: right shaded grey bar) grown on MM supplemented with 1 mM methionine. *CBS1* and *CGL1* are both up-regulated in *Δmet6* mutants. Gene expression was normalized relative to the level of the constitutive gene *ILV5* (MGG_15774.7). Each bar is the mean value of three replicates with standard deviations as vertical lines. Analyzed genes were: *MET6*, methionine synthase; *CBS1*, cystathionine *β*-synthase; *CGL1*, cystathionine *γ*-lyase. *Differences in expression are significant according to a t-test (5%).

**Fig 7:**
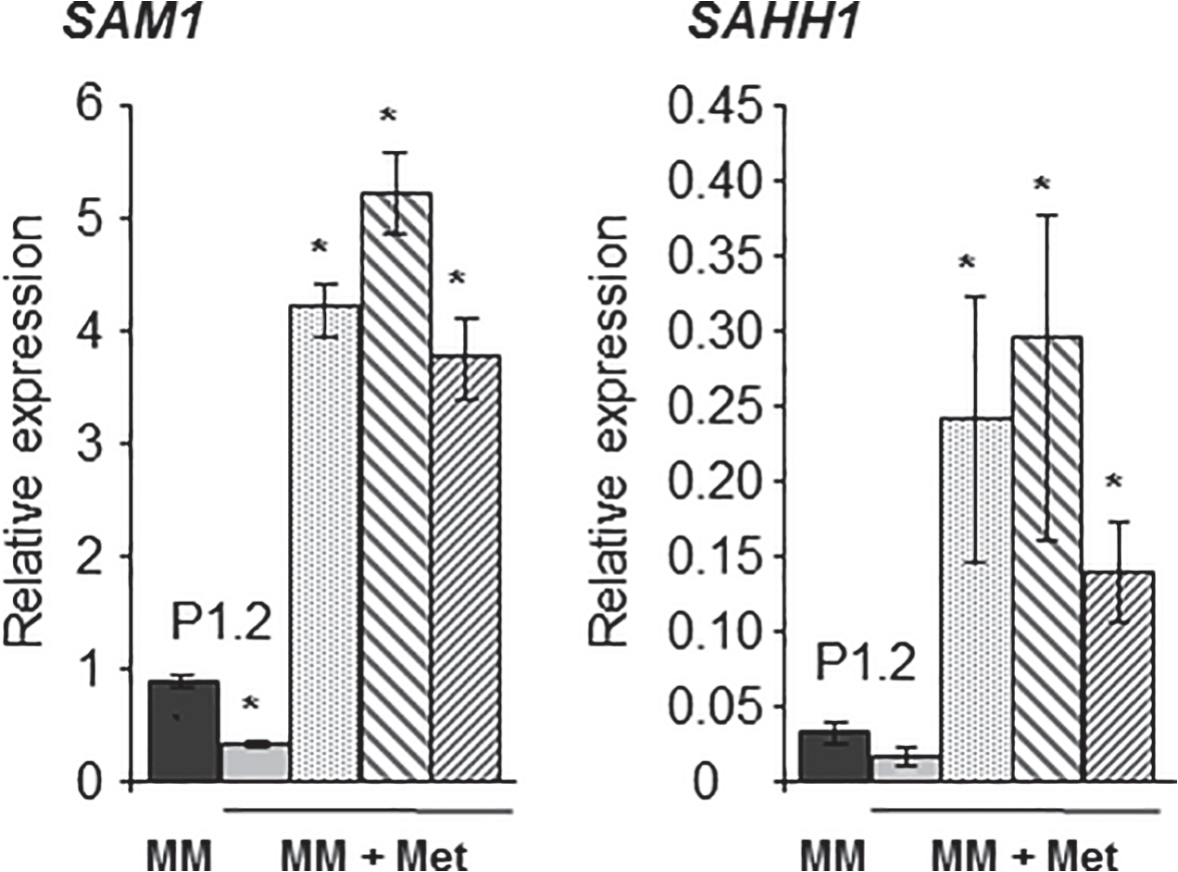
Expression profiles of genes from methyl cycle in *M*. *oryzae Δmet6* mutants and wild type. Transcript levels in mycelium of wild type P1.2 isolate grown in MM (MM: black bar). Transcript levels in mycelium of wild type P1.2 (grey bar) and three *Δmet6* mutants (M15.1: punctuated grey bar; M22.1: left shaded grey bar; and M23.1: right shaded grey bar) grown on MM supplemented with 1 mM methionine. *SAM1* and *SAH1* are strongly up-regulated in *Δmet6* mutants (10 to 16 fold). Gene expression was normalized relative to the level of the constitutive gene *ILV5* (MGG_15774.7). Each bar is the mean value of three replicates with standard deviations as vertical lines. Analyzed genes were: *SAM1*, *S*-adenosylmethionine synthetase and *SAHH1*, *S*-adenosylhomocysteine hydrolase. *Differences in expression are significant according to a t-test (5%).

The accumulation of homocysteine and cystathionine observed in *Δmet6* mutants likely influence the reverse transsulfuration pathway that metabolizes these coumpounds into cysteine. Two key enzymes are involved in this pathway (Fig 1). *Cbs*1 metabolizes homocysteine in the presence of serine into cystathionine, while *Cgl*1 metabolizes cystathionine into cysteine. We analyzed the expression of these two genes, *CBS1* and *CGL1*, in both wild type and *Δmet6* mutants. In wild type, addition of 1 mM methionine to MM did not modify the expression of *CBS1*, while the expression of *CGL1* was slightly up-regulated by 1.7-fold (Fig 6A). In *Δmet6* mutants grown on MM supplemented with 1 mM methionine, the expression of *CBS1* was much higher (5- to 8-fold, Fig 6B) than in wild type, while the expression of *CGL1* was only slightly up-regulated (1.5-fold, Fig 6B). These results suggest that *CBS1* is specifically up-regulated in *Δmet6* mutants. Expression of genes involved the methyl cycle, namely SAM synthetase (*SAM1*) and SAH hydrolase (*SAHH1*; Fig 1) was also assayed. BlastP search of *M*. *oryzae* genome using *S*. *cerevisiae* Sam1p (Ylr180p) or Sam2p (Ydr502cp) and Sah1p (Yer043cp) identified single hits respectively *MgSAM1* (MGG_00383.7) and *MgSAHH1* (MGG_05155.7). In wild type, addition of 1 mM methionine to MM down regulated the expression of *SAM1* by 2.7-fold, and *SAHH1* by 2-fold (Fig 7). In contrast, the expression of *SAM1* and *SAHH1* was up-regulated (10 to 15- folds Fig 7) in *Δmet6* compared to wild type.

## Discussion

### Nutritional requirements of *M*. *oryzae* methionine synthase deletion mutants

*M*. *oryzae* Met6 belongs to the family of cobalamine-independent methionine synthases (Fig 2). A genetic analysis of this metabolic pathway was perfomed in *M*. *oryzae* using *MET6* deletion mutants obtained by targeted gene replacement. The *Δmet6* mutants were unable to grow on MM (nitrate and sulfate as nitrogen and sulfur sources) unless supplemented with methionine (Fig 1; Table 1). The growth of the *Δmet6* mutant was completely blocked on MM (Fig 4A), suggesting that there are no alternative metabolic pathways for this type of mutant. These results demonstrate that Met6 is the only enzyme involved in the conversion of homocysteine into methionine *in M*. *oryzae*. This finding is in agreement with the fungal methionine biosynthetic pathway proposed for ascomycotina or basidiomycotina (*N*. *crassa*, [33]; *A*. *nidulans*, [34,]; *C*. *neoformans*, [35]; *F*. *graminearum*, [36, 37]; *U maydis*, [38]).

Other methionine auxotrophic mutants were described in *M*. *oryzae* such as *met1-*, which is probably defective for cystathionine gamma-synthase (Cgs1, [9]), and *str3-* defective for cystathionine beta-lyase (Cbl1, [10]), were leaky auxotrophs with some residual growth on MM. In *A*. *nidulans*, a alternative pathway involving homocysteine synthase (Hcs1, Fig 1) synthesizes homocysteine directly from *O*-acetylhomoserine and sulfide [12, 29, 34], independently of the biosysnthesis of homocysteine from cystathionine (Cbl1, Fig 1). This Hcs1 pathway could bypass the metabolic defects of *met1-* and *str3-* mutants, although at low efficiency, since these mutants are still strongly reduced in their growth on MM. In support of this hypothesis, we identified by BlastP search of *M*. *oryzae* protein database, a gene (MGG_07195.7) closely related to *A*. *nidulans* Hcs1 that encodes a protein with all the domains characteristic of homocysteine synthase. Further genetic analysis is needed to test if deletion of this gene in a *met1-* or a *str3-* background leads to double mutants that are as auxotrophic as our *Δmet6* mutants.

Alternative metabolic pathways leading to methionine biosynthesis from homocysteine independent of Met6 have been described in fungi. In *A*. *nidulans*, betaine and choline are metabolized into methionine by a betaine-homocysteine methyltransferase [28]. In *S*. *cerevisiae*, SAM and SMM are metabolized into methionine by two specific SAM- and SMM-homocysteine methyltransferases encoded by SAM4 and MHT1, respectively [31, 32]. Betaine and choline were unable to restore the growth of *M*. *oryzae Δmet6* mutants on MM, suggesting a defect in betaine-homocysteine methyltransferases or an inability to import betaine/choline compared to *A*. *nidulans*. SAM and SMM only partially restored the growth of *Δmet6* mutants on MM, suggesting the occurence of SAM- and SMM- homocysteine methyltransferases in *M*. *oryzae*. BlastP search of *M*. *oryzae* protein database using *S*. *cerevisiae* Sam4p and Mht1p protein sequences as queries, revealed a single hit (MGG_04215.7) with respectively 39% and 42% sequence similarity. This *M*. *oryzae* protein is good candidate for a SAM/SMM homocysteine methyltransferase. Further genetic and biochemical work are needed to test if this gene encodes a homocysteine methyltransferases involved in the conversion of SAM/SMM into methionine. No specific SAM or SMM transporters such as those described in yeast (SAM3 and MMP1, [31]) were identified in *M*. *oryzae* protein database.

### Sulfur amino-acids metabolism in *M*. *oryzae*: methionine homeostasis, homocysteine catabolism and SAM methyl cycle

Quantification of sulfur metabolites (Table 3) in wild type *M*. *oryzae* grown on different media showed that the intracellular concentration of methionine was maintained at a constant level of 1.6 μM (1.3–1.5 nmoles.mg^-1^ dry weight mycelium, Table 3), even on MM supplemented with excess exogenous methionine (1 mM). *M*. *oryzae Δmet6* mutant grown on MM supplemented with methionine also displays the same level of methionine as wild type (1.6 μM, Table 3). This methionine homeostasis is a physiological phenomenom not previously described in fungi. Indeed, feeding wild type *S*. *cerevisiae* yeast cells with exogenous methionine (0.5 mM) increased intracellular methionine levels up to 200-fold (100 mM) compared to levels on minimal medium (0.5 mM, [39]). Therefore, *M*. *oryzae* appears to have particular metabolic networks maintaining a constant level of intracellular methionine independently of its extracellular level or its genetic context (wild type, *Δmet6*). This homeostatis may reflect the importance of methionine as a substrate for metabolic pathways such as SAM-based methylation (secondary metabolites and DNA) and polyamine biosynthesis. The levels of homocysteine and cystathionine detected in *M*. *oryzae Δmet6* mutants grown on MM supplemented with methionine were much higher than those of wild type (10- to 13-fold, respectively, Table 3). Similar trends were observed in other fungal methionine synthase mutants. In *A*. *nidulans METH* null mutants accumulated 2-fold cystathionine compared to wild type [28]. In *S*. *pombe*, methionine synthase mutants accumulate 18-fold homocysteine compared to wild type [40]. This accumulation is reduced to 7-fold by the introduction of *S*. *cerevisiae* genes involved in the reverse transsulfuration pathway that are lacking in *S*. *pombe*, demonstrating the importance of this pathway in metabolizing excess homocysteine [40].

In *M*. *oryzae*, we found that transcript levels of *CBS1* from the reverse transulfuration pathway (Cbs1, Fig 1) was much higher in *Δmet6* (5- to 8-fold, Fig 6B) than in wild type. Similar observations were also reported in *A*. *nidulans* treated with high concentrations of exogenous homocysteine or methionine [41]. This up-regulation probably leads to an increase in the metabolisation of homocysteine into cysteine. However, this response is not sufficient to avoid homocysteine accumulation in *Δmet6*. The reverse transulfuration pathway is also important in wild type for metabolizing exogenous homocysteine that is toxic over 0.2 mM (S4 Fig). Indeed, previous studies have shown that the deletion of *CBS1* [30] strongly increases the toxicity of homocysteine to *M*. *oryzae*. In addition, the toxicity of homocysteine to *M*. *oryzae* is partially reversed by the addition of serine that is a co-susbtrate needed for the transformation of homocysteine into cystathionine (Fig 1, S4 Fig), suggesting that this amino-acid is a limiting factor in this metabolic process.

Another major modification observed in *Δmet6* mutants compared to wild type, is an increase in SAM and SAH levels (3- to 5-fold, respectively) coupled to a strong up-regulation (12- to 17-fold) of *SAHH1* and *SAM1*. To our knowledge, this type of up-regulation in a *MET6* null mutant was not yet described in eukaryotes. These modifications are not a consequence of methionine feeding, as wild type isolate displayed a reduction in *SAM1*and *SAHH1* expression when treated with methionine (Fig 7). The increase in SAM and SAH levels in *Δmet6* could result from a physiological response to its metabolic defect. The up-regulation of *SAM1* in *Δmet6* mutants could explain the increase in SAM observed in this mutant. However, the metabolic consequences of the up-regulation of *SAHH1* are more difficult to interpret in the context of *Δmet6*. One possible hypothesis is that high levels of cellular SAH triggers the expression of *SAHH1* to accelerate its transformation into homocysteine. Overall, the *SAM1* and *SAHH1* trasncriptional responses observed in *Δmet6* suggests that they are additional candidates for the homocysteine regulon reported in *A*. *nidulans* [41].

### Pathogenicity defects of *M*. *oryzae* methionine synthase deletion mutants

*M*. *oryzae Δmet6* mutants were non-pathogenic on intact and wounded barley and rice leaves. These mutants differentiated appressoria of normal appearance but these were unable to penetrate into intact host leaves (S7 Fig). The penetration defect of *Δmet6* suggests that appressoria have a high requirement for methionine. In support of this hypothesis, we observed that *MET6* was up regulated in appressoria compared to spores (10-fold, Fig 3). This induction of methionine synthase encoding genes was also observed in appressoria from other plant pathogenic fungi [8, 10]. Indeed, methionine and its derivative SAM could be involved in the methylation of important appressorial secondary metabolites such as DHN, the precursor of melanin [42]. DHN-melanin is essential for appressorium-mediated penetration and the penetration defects of DHN-melanin deficient mutants are rescued by leaf wounding [1, 2]. However, *Δmet6* mutants did not infect wounded leaves, showing that their reduced melaninization is not responsible of their defect in pathogenicity. The unablity to infect intact and wounded leaves of *Δmet6* is more probably caused by the absence of free methionine in host plant tissues and apoplastic fluids [8]. Indeed, the nutritional complementation of *Δmet6* pathogenicity defects by exogenous methionine clearly establishes that these mutants cannot assimilate methionine from the plant. This also hold true for SMM which complements the pathogenicity defects of *Δmet6* (S6 Fig). These results suggest that rice and barley SMM, known to accumulate to high levels in leaf cell vacuoles [43, 44], is not available for *M*. *oryzae* during infection, likely as a result of its strict localization into the plant vacuole.

The three other *M*. *oryzae* methionine auxotrophic mutants already described (*met1-*, *str3-* and *met13-*) displayed a strong reduction in pathogenicity on intact barley and rice leaves (lower number of lesions and reduced lesion size [9, 10, 11]). For example, *str3-* is able to penetrate into rice leaves and to slowy develop infectious hyphae into infected rice cells leading to the formation of very small lesions [10]. Since *M*. *oryzae Δmet*6 mutants are non-pathogenic on rice and barley leaves and are unable to penetrate into host leaves, the quantitative pathogenicity defects of *met1-*, *str3-* and *met13-* mutants suggest that they are not completely blocked either in their methionine biosynthesis or in rescuing reduced sulfur sources from the plant. Indeed, an alternative metabolic pathway for homocysteine biosynthesis (Hcs1, Fig 1) is likely responsible for bypassing the metabolic defects of *met1-* and *str3-*. This bypass may explain their reduced pathogenicity compared to the full non pathogenicity of *Δmet6* that is the only available mutant unable to synthesize methionine during infection.

Methionine auxotrophic mutants have been also described other plant pathogenic fungi. Methionine auxotrophic mutants from the corn smut fungus *U*. *maydis* were reduced in pathogenicity on corn leaves [38, 45]. These mutants were able to penetrate and colonize host tissues, although to a lesser extent than wild type and produced fewer and smaller tumors. These results suggest that *U*. *maydis MET6* mutants were able to acquire sufficient amounts of methionine or its derivatives from maize tissues to support its growth, but not tumor formation. Similarly, methionine synthase mutants from the wheat fungal pathogen *F*. *graminearum* displayed a highly reduced pathogenicity on wheat heads [36]. These findings suggest that the requirement for methionine and its derivatives may differ among plant pathogenic fungi, likely as a results of their ability to assimilate methionine or its derivatives from specific host tissues (roots, vascular tissue, leaf cells, apoplast, grains).

## Supporting Information

S1 Fig *M*. *oryzae MET6* locus in *Δmet6* and wild type. **A**- The three exons of *MET6* gene are shown as hatched boxes separated by introns. Partial 5’UTR and 3’UTR are indicated as grey lines under the gene. **B**- Construction of *MET6* gene replacement vector. 1 kb genomic regions (Left Border and Right Border) flanking *MET6* ORF indicated as grey boxes were amplified using primers (arrows, see S1A Table). *MET6* exons are shown as hatched boxes separated by introns. **C**- Structure of *MET6* locus in *Δmet6* mutants. Dark boxes represent hygromycin resistance cassette. Grey boxes correspond to Left Border and Right Border sequences flanking *MET6* (see B).
(TIF)

S2 Fig Southern blot analysis of *M*. *oryzae* wild type, *Δmet6* mutants and an ectopic transformant. Agarose gel electrophoresis of *M*. *oryzae* genomic DNA digested with *Bam*HI. *MET6* right border (RB) was amplified using *MET6*-3 and *MET6*-4 primers and used as a probe for Southern hybridization. *Δmet6* P1.2 mutants (M4.1, M15.1, M22.1, M23.1) and one ectopic P1.2 transformant (E19.1) are displayed. *Δmet6* has a *BamH*1 fragment of 3.3 Kb, while wild type and ectopic transformant E19.1 displayed a 8.1 Kb BamH1 fragment. Additional band observed for ectopic transformant E19.1 corresponds to vector integration at another location than *MET6*.
(TIF)

S3 Fig Alignement of Met6 from *Magnaporthe oryzae* (MGG_06712.7) with known fungal and plant methionine synthases. *Neurospora crassa* (AF404820_1), *Aspergillus nidulans* (AF275676_1), *Saccharomyces cerevisiae* (Yer091cp) and *Arabidopsis thaliana* (AT3G3) protein sequences were aligned with ClustalW and conserved amino-acids were highlighted with BoxShade. Conserved amino-acids of the C-terminal domain involved in the binding of zinc (*), of homocysteine ($) and of the pterin moiety of methyl-triglutamate-tetrahydrofolate (¤) according to *Arabidopsis thaliana* cobalamin-independent methionine synthase crystal structure (PDB entry: 1U1J, 1U1H, 1U1U and 1U22) [27].
(DOC)

S4 Fig Effect of homocysteine on growth of *M*. *oryzae*. Wild type *M*. *oryzae* P1.2 was grown on MM supplemented with homocysteine (HCys, 0.05 to 2 mM). At concentrations higher than 0.1 mM HCys, a significant growth inhibition was observed that was partially reversed by 1 mM serine (Ser).
(TIF)

S5 Fig Growth profiles of *M*. *oryzae* wild type, *Δmet6*, ectopic transformant E12.1, and *Δmet6*::*MET6* on methionine and its derivatives. Wild type isolate P1.2 (Wild type P1.2), *Δmet6* M15.1 (*Δmet6* M15.1), ectopic transformant E12.1 (Ectopic E12.1), and *Δmet6*::*MET6* complemented transformant 1.2 (Complemented *Δmet6* 1.2) were grown on MM supplemented or not with methionine and its derivatives at a final concentration of 1 mM: Met, methionine; SMM, *S*-methylmethionine; SAM, *S*-adenosylmethionine; MTA, methylthioadenosine (1% dimethylsulfoxide final). Observations were performed 5 days after plate inoculation.
(TIF)

S6 Fig Pathogenicity of *M*. *oryzae Δmet6* mutants on barley leaves in presence of methionine and its derivatives. Barley cv. Express leaves were inoculated with spore suspension droplets of *M*. *oryzae* Wild type isolate P1.2 (Wild type P1.2), *Δmet6* M15.1 (*Δmet6* M15.1), ectopic transformant E12.1 (Ectopic E12.1), and *Δmet6*::*MET6* complemented transformant 1.2 (Complemented *Δmet6* 1.2) in the presence of methionine and its derivatives: Met, methionine; SMM, *S*-methylmethionine; SAM, *S*-adenosylmethionine; MTA, methylthioadenosine (1% dimethylsulfoxide final).
(TIF)

S7 Fig Cytological observations of early stages of infection of onion epidermis by *M*. *oryzae* wild type and *Δmet6*. Spores of wild type P1.2 isolate (P1.2 wild type) and *Δmet6* M15.1 (*Δmet6*) were treated with methionine (1 mM, + Met) or not, and deposited on detached onion epidermis. Epidermal layer was stripped 24–30 h after inoculation. Appressoria formed onion epidermis were stained with Cotton blue in lactic acid and observed using a bright field microscope. Penetration was scored as successful if unstained infectious hyphae were detected inside epidermal cells underneath appressoria. Ap, appressorium; Hy, infectious hyphae; Sp, spores.
(TIF)

S1 Table List of primers (DOCX)

S2 Table Sequences used to construct S2 Fig. (DOCX)
