# Supplementary figures and images for: Methionine Biosynthesis is Essential for Infection in the Rice Blast Fungus Magnaporthe oryzae
Source: PLoS One. 2015 Apr 9;10(4):e0111108. doi: 10.1371/journal.pone.0111108 (PMC4391826; doi:10.1371/journal.pone.0111108)

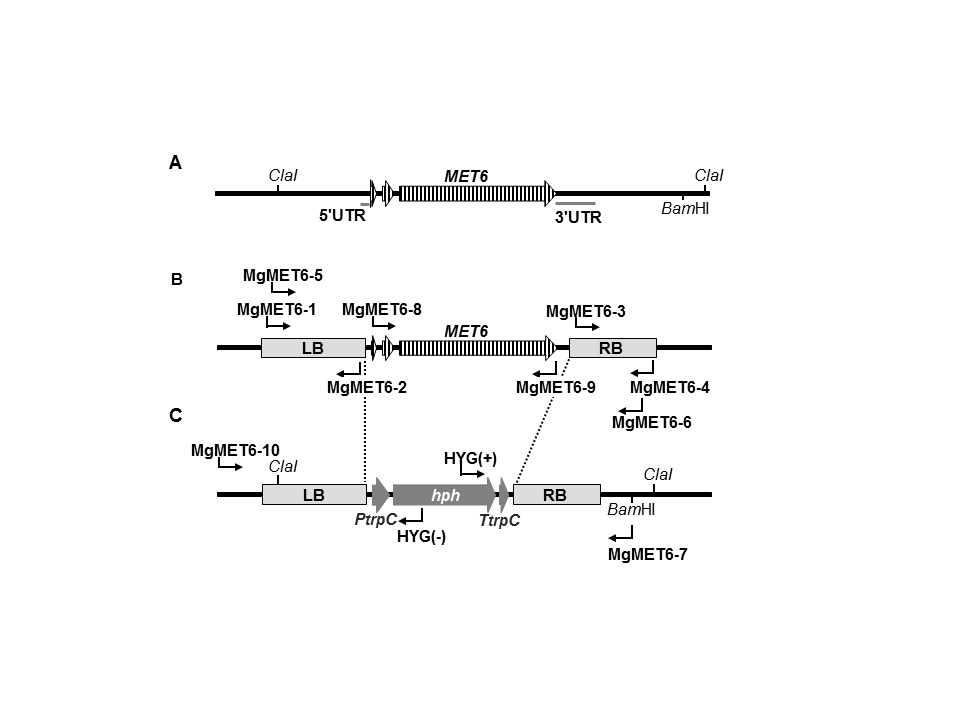

Supplement: S1 Fig — A- The three exons of MET6 gene are shown as hatched boxes separated by introns. Partial 5’UTR and 3’UTR are indicated as grey lines under the gene. B- Construction of MET6 gene replacement vector. 1 kb genomic regions (Left Border and Right Border) flanking MET6 ORF indicated as grey boxes were amplified using primers (arrows, see S1A Table). MET6 exons are shown as hatched boxes separated by introns. C- Structure of MET6 locus in Δmet6 mutants. Dark boxes represent hygromycin resistance cassette. Grey boxes correspond to Left Border and Right Border sequences flanking MET6 (see B). (TIF) [file pone.0111108.s001.tif]

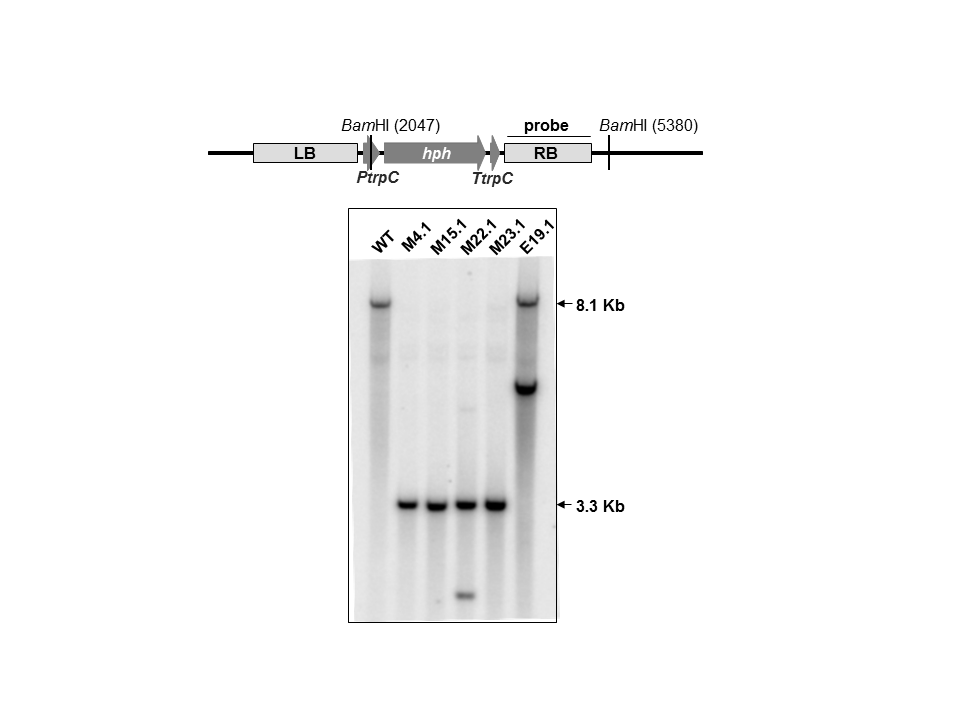

Supplement: S2 Fig — Agarose gel electrophoresis of M. oryzae genomic DNA digested with BamHI. MET6 right border (RB) was amplified using MET6-3 and MET6-4 primers and used as a probe for Southern hybridization. Δmet6 P1.2 mutants (M4.1, M15.1, M22.1, M23.1) and one ectopic P1.2 transformant (E19.1) are displayed. Δmet6 has a BamH1 fragment of 3.3 Kb, while wild type and ectopic transformant E19.1 displayed a 8.1 Kb BamH1 fragment. Additional band observed for ectopic transformant E19.1 corresponds to vector integration at another location than MET6. (TIF) [file pone.0111108.s002.tif]

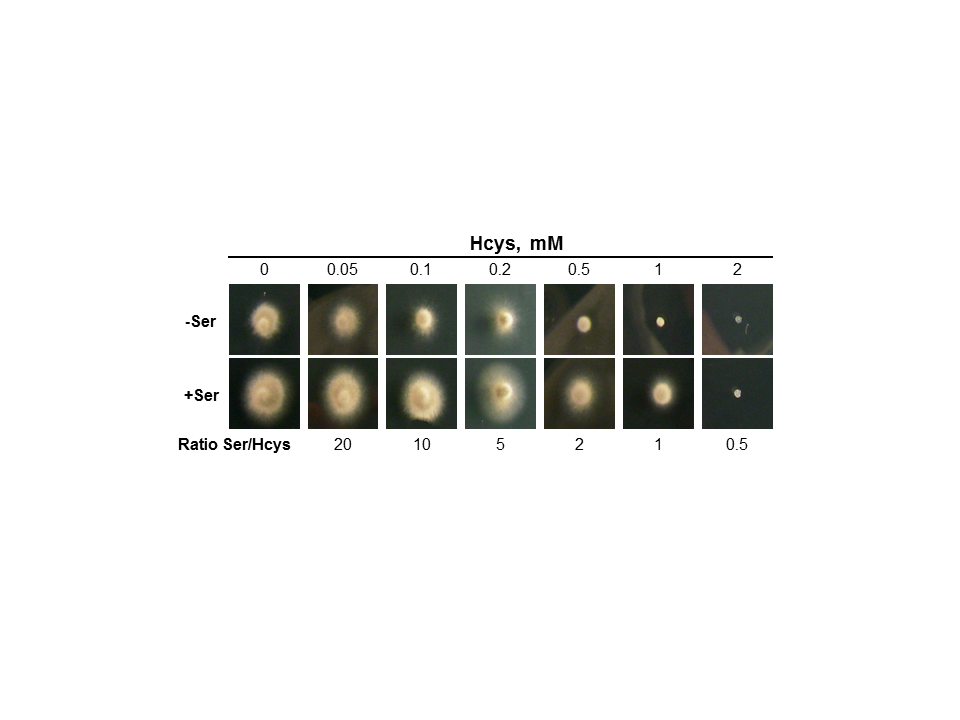

Supplement: S4 Fig — Wild type M. oryzae P1.2 was grown on MM supplemented with homocysteine (HCys, 0.05 to 2 mM). At concentrations higher than 0.1 mM HCys, a significant growth inhibition was observed that was partially reversed by 1 mM serine (Ser). (TIF) [file pone.0111108.s004.tif]

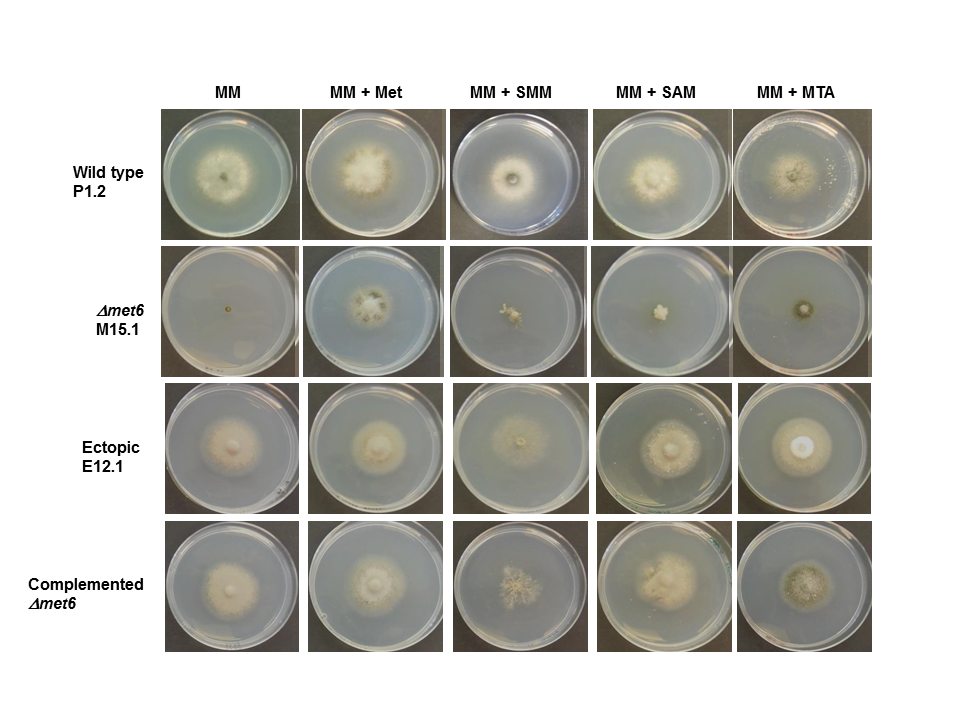

Supplement: S5 Fig — Wild type isolate P1.2 (Wild type P1.2), Δmet6 M15.1 (Δmet6 M15.1), ectopic transformant E12.1 (Ectopic E12.1), and Δmet6::MET6 complemented transformant 1.2 (Complemented Δmet6 1.2) were grown on MM supplemented or not with methionine and its derivatives at a final concentration of 1 mM: Met, methionine; SMM, S-methylmethionine; SAM, S-adenosylmethionine; MTA, methylthioadenosine (1% dimethylsulfoxide final). Observations were performed 5 days after plate inoculation. (TIF) [file pone.0111108.s005.tif]

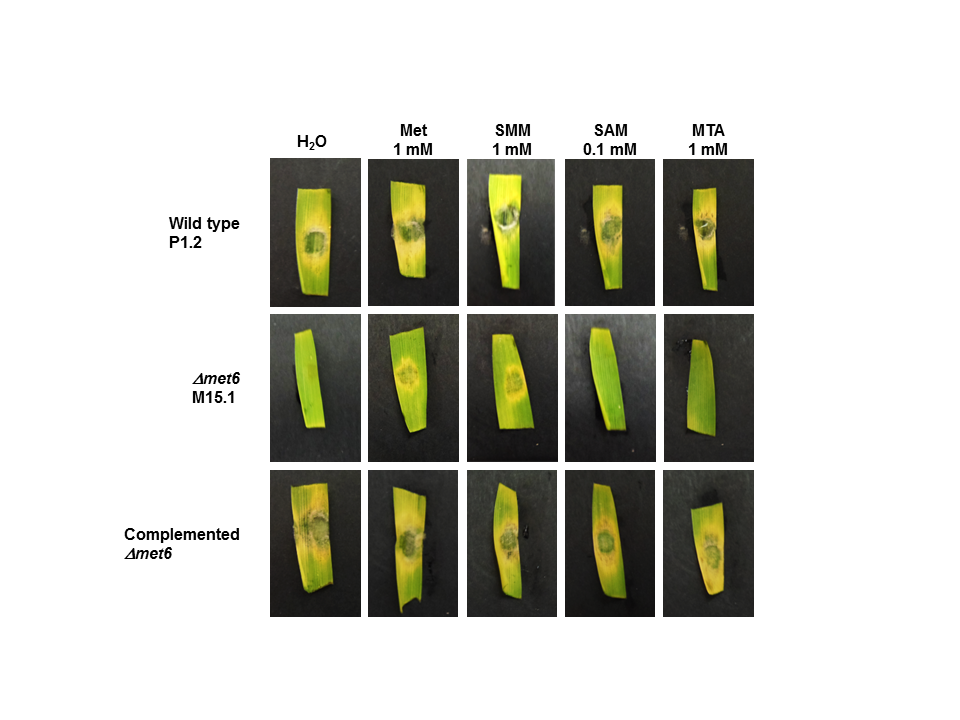

Supplement: S6 Fig — Barley cv. Express leaves were inoculated with spore suspension droplets of M. oryzae Wild type isolate P1.2 (Wild type P1.2), Δmet6 M15.1 (Δmet6 M15.1), ectopic transformant E12.1 (Ectopic E12.1), and Δmet6::MET6 complemented transformant 1.2 (Complemented Δmet6 1.2) in the presence of methionine and its derivatives: Met, methionine; SMM, S-methylmethionine; SAM, S-adenosylmethionine; MTA, methylthioadenosine (1% dimethylsulfoxide final). (TIF) [file pone.0111108.s006.tif]

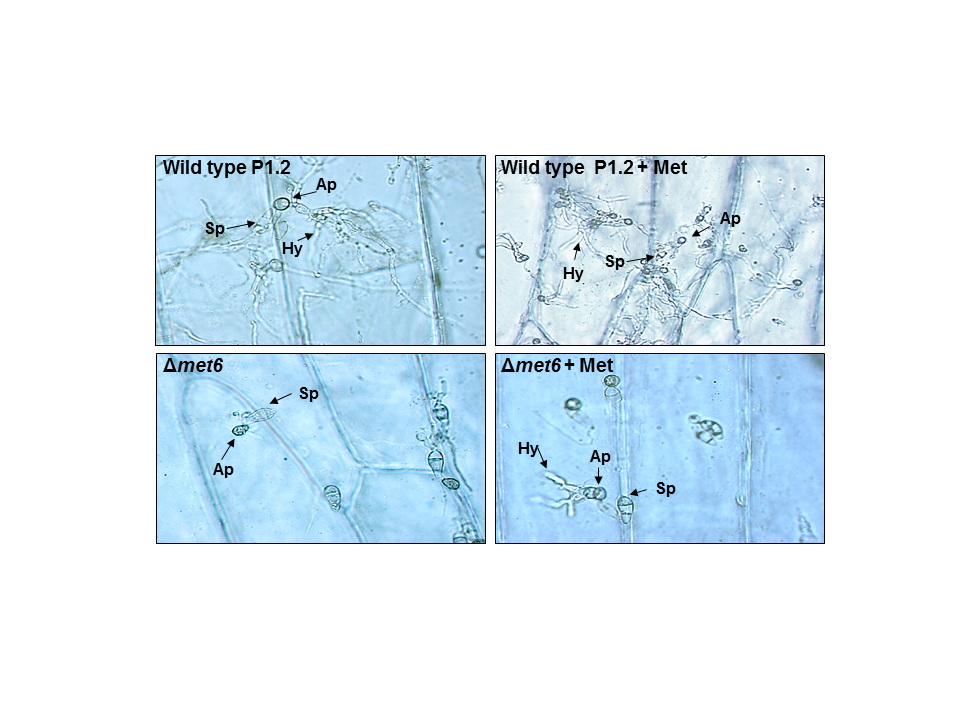

Supplement: S7 Fig — Spores of wild type P1.2 isolate (P1.2 wild type) and Δmet6 M15.1 (Δmet6) were treated with methionine (1 mM, + Met) or not, and deposited on detached onion epidermis. Epidermal layer was stripped 24–30 h after inoculation. Appressoria formed onion epidermis were stained with Cotton blue in lactic acid and observed using a bright field microscope. Penetration was scored as successful if unstained infectious hyphae were detected inside epidermal cells underneath appressoria. Ap, appressorium; Hy, infectious hyphae; Sp, spores. (TIF) [file pone.0111108.s007.tif]
