# Supplementary material for: Methionine Biosynthesis is Essential for Infection in the Rice Blast Fungus Magnaporthe oryzae
Source: PLoS One. 2015 Apr 9;10(4):e0111108. doi: 10.1371/journal.pone.0111108 (PMC4391826; doi:10.1371/journal.pone.0111108)
Supplement: S3 Fig — Neurospora crassa (AF404820_1), Aspergillus nidulans (AF275676_1), Saccharomyces cerevisiae (Yer091cp) and Arabidopsis thaliana (AT3G3) protein sequences were aligned with ClustalW and conserved amino-acids were highlighted with BoxShade. Conserved amino-acids of the C-terminal domain involved in the binding of zinc (*), of homocysteine ($) and of the pterin moiety of methyl-triglutamate-tetrahydrofolate (¤) according to Arabidopsis thaliana cobalamin-independent methionine synthase crystal structure (PDB entry: 1U1J, 1U1H, 1U1U and 1U22) [27]. (DOC) [file pone.0111108.s003.doc]

**Magnaporthe 1 MVQSAILGFPRMGVNRDLKKATEAYWAGKISQDDLLAEAKRLRLAHWKIQKDAGVDIIPSNDFALYDQVLSHIQDFGAVP
Neurospora 1 MVQSSVLGFPRMGVLRDLKKANEAYWADKISQEALLAEGKRLRLAHWKIQKDAGVDIIPSNDFAHYDHVLDHIQLFNAVP
Aspergillus 1 MVQSAVLGFPRMGKLRDLKKATEAYWGEKISRDELLSEGKRLRAEHWKIQKDAGVDIIPSNDFAFYDQVLDHIQLFGVVP
Saccharomyces 1 MVQSAVLGFPRIGPNRELKKATEGYWNGKITVDELFKVGKDLRTQNWKLQKEAGVDIIPSNDFSFYDQVLDLSLLFNVIP
Athaliana3G03 386 -MASHIVGYPRMGPKRELKFALESFWDGKSSADDLQKVSADLRSDIWKQMSAAGIKYIPSNTFSHYDQVLDTTAMLGAVP


Magnaporthe 81 ERYSSS-KLNPVDEYFAMGRGHQKD------GVDVPSLEMVKWFDSNYHYVKPTLQDNQTFTLTANPKAVNEFNEAKEAG
Neurospora 81 ERYTSQ-KLSPLDEYFAMGRGHQKG------GVDVPALEMVKWFDSNYHYVKPTLQDNQTFSLAKDPKPVREFLEAKEAG
Aspergillus 81 ERYSKY-NLHPLDEYFAMGRGLQKPAKDGQPAIDVPSLEMVKWFDSNYHYVKPTLQDNQTFKLAANPKPVVEFLEAKEAG
Saccharomyces 81 DRYTKY-DLSPIDTLFAMGRGLQRKATETEKAVDVTALEMVKWFDSNYHYVRPTFSKTTQFKLN-GQKPVDEFLEAKELG
Athaliana3G03 386 SRYGFTSGEIGLDVYFSMARGN----------ASVPAMEMTKWFDTNYHYIVPELGPEVKFSYA-SHKAVNEYKEAKALG


Magnaporthe 154 INTRPVLVGPVSFLHLAKADRGQ--SVDPIDLLDKLVPVYEELLAKLKAAGAETVQIDEPVLVFDLPAKVKAAFKPTYEK
Neurospora 154 FQTRPVLVGPVSFLALGKADRGS--SVDPITLLDKLVPVYVELLKQLKAAGAESVQIDEPVLVFDLRPEVKAAFKPAYEA
Aspergillus 160 IVTRPVILGPVSFLTLAKADRGQ--TLDPISKIDELLPLYVELLTKLKEAGVEDVQIDEPVLVFDLPLKSKNAFKPAYEK
Saccharomyces 159 IHTRPVLLGPVSYLFLGKADKDS-LDLEPLSLLEQLLPLYTEILSKLASAGATEVQIDEPVLVLDLPANAQAAIKKAYTY
Athaliana3G03 386 VETVPVLVGPVSYLLLSKLAKGVDKSFDLLSLLPKILPVYKEVIAELKAAGASWIQLDEPLFVMDLEGHKLQAFSGAYAE


Magnaporthe 232 FASLGDKIPKLVFATYFGDIVH-NLDLVPK--DVYAVHVDLVRNPEQLETVVGALGPKTILSAGIVDGRNIWKTNFQKAI
Neurospora 232 IAAAGDAVPKVVVATYFGDIVH-NFDVLPAFSGAAGLHVDLVRNPEQLEPVLKQLGPNQILSAGVVDGRNIWKNDFAKSL
Aspergillus 238 LGSLGAQAPRLVLATYFGDIVH-NIDVLPALHNIYGIHIDLVRNPEQLDSVIGALGPKQVLSAGVVDGRNIWKTNFKAAI
Saccharomyces 238 FGEQ-SNLPKITLATYFGTVVP-NLDAIKGL-PVAALHVDFVRAPEQFDEVVAAIGNKQTLSVGIVDGRNIWKNDFKKSS
Athaliana3G03 386 LESTLSGL-NVLVETYFADIPAEAYKTLTSLKGVTAFGFDLVRGTKTIDLIKSGFPQGKYLFAGVVDGRNIWANDLAASL


Magnaporthe 309 ETVESAIQKLGKERVIVATSSSLLHTPHTLASEKKLDPEIADWFSFASEKAVEVAIIAKAVTEGPAAVREQLEANAKSMN
Neurospora 311 EILQTAVKALGSERVIVATSSSLIHTPHTLASEKKLPSDVYEWFSFAVEKVKEVATLAKAVT-EPEAVKAELEANAAAIK
Aspergillus 317 EKVELAIQKLGKDRVIVSTSSSLLHVPHTLASEKNLDPEVQDWFSFAVEKTSEVVVIAKAVTEGPAAVREQLEANAKSVQ
Saccharomyces 315 AIVNKAIEKLGADRVVVATSSSLLHTPVDLNNETKLDAEIKGFFSFATQKLDEVVVITKNISGQ--DVAAALEANAKSVE
Athaliana3G03 386 ITLQSLEGVVGKDKLVVSTSCSLLHTAVDLINETKLDAEIKSWLAFAAQKVVEVDALAKALAGQ--TNESFFTANADALS

 $ $**

**Magnaporthe 389 ARATSSRTNDPKVKERQSKIVESDYNRKSEFPTRISQQQAKLNLPLFPTTTIGSFPQTQTIRAQRAKLTKKEIDAEQYAK
Neurospora 390 ARTDSKRTNDPAVKERQAQVTPEQHNRKAPFNTRYAEQKKHLSLPLFPTTTIGSFPQTSEIRVQRNKFTKGEISAEEYER
Aspergillus 397 ARASSKRTNDPKVKERQAAVTPEQHNRKSPFPVRIAEQTKSIKLPLFPTTTIGSFPQTKEIRIQRNKFTKGEITAEEYEK
Saccharomyces 393 SRGKSKFIHDAAVKARVASIDEKMSTRAAPFEQRLPEQQKVFNLPLFPTTTIGSFPQTKDIRINRNKFNKGTISAEEYEK
Athaliana3G03 386 SRRSSPRVTNESVQKAAAALKGSDHRRTTEVSARLDAQQKKLNLPILPTTTIGSFPQTVELRRVRREYKAKKISEEDYVK**

**Magnaporthe 469 FIEEEIENNVKIQEELGLDVFVHGEPERNDMVQFFGERLDGYAFTTHAWVQSYGSRCVRPPIIVGDISRPAPMTVKESRY
Neurospora 470 FIEKEIELAVKIQDELDLDVYVHGEPERNDMVQYFGERLNGYVFTTHAWVQSYGSRCVRPPIIVGDISRPAPMTVKESKY
Aspergillus 477 FIEKEIAEVVKIQEELGLDVLVHGEPERNDMVQYFGERLTGYVFTTHAWVQSYGSRCVRPPIIVGDISRPAPMTVKESKY
Saccharomyces 473 FINSEIEKVIRFQEEIGLDVLVHGEPERNDMVQYFGEQINGYAFTVNGWVQSYGSRYVRPPIIVGDLSRPKAMSVKESVY
Athaliana3G03 386 AIKEEIKKVVDIQEDLDIDVLVHGEPERNDMVEYFGEQLSGFAFTANGWVQSYGSRCVKPPVIYGDVSRPKPMTVFWSST

 ¤ $
Magnaporthe 549 AVEISKKPMKGMLTGPVTCLRWSFPRDDVHQSVQAEQLALALRDEVVDLEKAGVDVIQVDEPALREGLPLRSGKERDAYL
Neurospora 550 AASISKKPMKGMLTGPVTCLRWSFPRVDVHQSVQCQQLALALRDEVVDLEKNGIYVIQVDEPALREGLPLRKGQEREAYL
Aspergillus 557 AVSISSKPMKGMLTGPITCLRWSFPRDDVHQSVQAQQLALALRDEVVDLEAAGVKVIQVDEPALREGLPLRAGKEREDYL
Saccharomyces 553 AQSITSKPVKGMLTGPITCLRWSFPRDDVDQKTQAMQLALALRDEVNDLEAAGIKVIQVDEPALREGLPLREGTERSAYY
Athaliana3G03 386 AQSMTKRPMKGMLTGPVTILNWSFVRNDQPRHETCYQIALAIKDEVEDLEKGGIGVIQIDEAALREGLPLRKA-EHSFYL

 * *
Magnaporthe 629 QWAVKAFKLSTCGVEDSTQIHSHFCYSEFQDFFHAIAALDADVLSIENSKSDAKLLKVFVD-SAYPRHIGPGVYDIHSPR
Neurospora 630 KWAVDSFKLATAGVENSTQIHSHFCYSEFQDFFHAIAALDADVLSIENSKSDAKLLKVFID-EEYPRHIGPGVYDIHSPR
Aspergillus 637 QWAVAAFRLSTSGVSDGTQIHSHFCYSEFQDFFHAIAALDADVLSIENSKSDAKLLKVFID-EAYPRHIGPGVYDIHSPR
Saccharomyces 633 TWAAEAFRVATSGVANKTQIHSHFCYSDLDP--NHIKALDADVVSIEFSKKDDANYIAEF--KNYPNHIGLGLFDIHSPR
Athaliana3G03 386 DWAVHSFRITNCGVQDSTQIHTHMCYSNFNDIIHSIIDMDADVITIENSRSDEKLLSVFREGVKYGAGIGPGVYDIHSPR

 *
Magnaporthe 708 VPSEQEIKDRIEEMLQYLKPEQLWIDPDCGLKTRQWKETKEALTNMVNAAKYFRAKYAK--
Neurospora 709 VPTLEEFKQRIEEMLAYLKPEQLWINPDCGLKTRKWDEVKGALSHMVEAAKYFREKYANKA
Aspergillus 716 VPSEQEIKDRVEEMLAYLRPEQLWINPDCGLKTRQWPETKAALSNLVQAAKYFREKYAK--
Saccharomyces 709 IPSKDEFIAKISTILKSYPAEKFWVNPDCGLKTRGWEETRLSLTHMVEAAKYFREQYKN--
Athaliana3G03 386 IPSTDEIADRINKMLAVLEQNILWVNPDCGLKTRKYTEVKPALKAMVDAAKLIRSQLGSAK**
