# Supplementary material for: Methionine Biosynthesis is Essential for Infection in the Rice Blast Fungus Magnaporthe oryzae
Source: PLoS One. 2015 Apr 9;10(4):e0111108. doi: 10.1371/journal.pone.0111108 (PMC4391826; doi:10.1371/journal.pone.0111108)
Supplement: S1 Table — (DOCX) [file pone.0111108.s008.docx]

**Table S1. List of primers**

**A. Primers used for vectors and locus analyses (see Supplementary Figure 1)**

| Primer | RS | Comment | Sequence (5’ to 3’) |
| --- | --- | --- | --- |
| MET6-1 MET6-2 MET6-3 MET6-4 MET6-5 MET6-6 MET6-7 MET6-8 MET6-9 MET6-10 HYG (+) HYG (-) | *EcoR* I *Sac*II | Left border Left border Right border Right border VML probe VML probe  VML PCR  VML *MET6* PCR  VML *MET6* PCR  Comp *MET6* PCR  HYG probe  HYG probe | CGGAATTCTGACAGTCTGCAATCGGAGGCTCCCCGCGGTGGACGGCTTCGGTGACTGGG  GAAGATCTAGTGCAAGCGATAAGTCTCCG GCGGGATCCGCATGGCTCCTCCTGCTAGGC GACAGTCTGCAATCGGAGGC GCATGGCTCCTCCTGCTAGGC GGCTCCGTTCCCAGCAATGC GTTCAATCAGCGATTCTCG GCTTATTTGGCGTACTTGGC GGAGTTGCTCAAGATATCGC TAGAGTAGATGCCGACCGGG AGGCTCTCGCTGAACTCCCCAATG |

TGR, targeted gene replacement vector; VML, verification of MET6 locus by PCR; Comp, MET6 complementation vector; HYG, hygromycine gene.

**B. Primers used for real time PCR**

| BROAD | Gene : Protein encoded | Primer sequence (5’ to 3’) |
| --- | --- | --- |
| MGG_06712.7 | *MET6*: Methionine synthase | Fw: CGTAACGACATGGTGCAATTCT Rv: CAGGCGTGCGTGGTGAA |
| MGG_07384.7 | *CBS1*: Cystathionine beta-synthase | Fw: CTCGACGTGCTCAGCAAGTTC Rv: TCGCCGCTCTTCTCAGTGA |
| MGG_10380.7 | *CGL1*: Cystathionine gamma-lyase | Fw: CACGCGTGTCTTCACATTGG Rv: TCATGACGCTGGGCAACTC |
| MGG_00383.7 | *SAM1*: SAM synthetase | Fw: CCAGGGTGACGCTGGACTT Rv: CCCAGCCACCGTACGT |
| MGG_05155.7 | *SAHH1*: SAH hydrolase | Fw: AGTCGCTTGTTGACGGTATCAA Rv: AGCGACAACGGCAATCTTG |
| MGG_15774.7 | *ILV5*: ketol-acid reductoisomerase | Fw: CCAGCTCTACGACTCGGTCAA Rv: AGTCGGGCTGGCTGTTGTAGT |
| MGG_03982.7 | *ACT1*: gamma actin | Fw: GGCACCGTCGTCGATGA Rv: CCACCGATCCAGACGGAGTA |

Fw: forward primers and Rv: reverse primers
