# Supplementary material for: Methionine Biosynthesis is Essential for Infection in the Rice Blast Fungus Magnaporthe oryzae
Source: PLoS One. 2015 Apr 9;10(4):e0111108. doi: 10.1371/journal.pone.0111108 (PMC4391826; doi:10.1371/journal.pone.0111108)
Supplement: S2 Table — (DOCX) [file pone.0111108.s009.docx]

**Table S2. Sequences used to construct Figure S2.**

**Ascomycota, A-Saccharomycotina,** ***Candida* sp.**: *Candida albicans* WO-1, CAWG_01512.1; *Clavispora lusitaniae* ATCC 42720, CLUG_00395.1; *Candida tropicalis* MYA-3404, CTRG_00928.3; *Lodderomyces elongisporus* NRRL YB-4239, LELG_00202.1; *Debaryomyces hansenii* CBS767, DEHA0A0752g; *Candida parapsilosis*, CPAG_00445; *Pichia guilliermondii* ATCC 6260, PGUG_02101.1; *Pichia pastoris*, AAT11796.1; *Pichia stipitis* CBS 6054, Picst3_78051; ***Saccharomyces* spp.**: *Ashbya gossypii*, AGOS_ABR212c; *Candida glabrata*, CAG_60404.1; *Kluyveromyces lactis* NRRL Y-1140, KLLA0E20163g; *Saccharomyces cerevisiae*, YER091c; *Yarrowia lipolytica* CBIB122, YALI0E12683g; **B-Taphrinamycotina**: *Schizosaccharomyces japonicus* yFS275, SJAG_02290.1; *Schizosaccharomyces octosporus*, SOCG_02338.2; *Schizosaccharomyces pombe* 972h-, SPAC9_09; **C-Pezizomycotina**: ***Aspergillus* spp.** : *Aspergillus clavatus* NRRL1, ACLA_046680; *Aspergillus flavus* NRRL3357, AFL2G_04650.2; *Aspergillus fumigatus* Af293, Afu4g07360; *Aspergillus nidulans* FGSC A4, AN44431.3; *Aspergillus niger* ATCC 1015, Aspni_203669; *Aspergillus oryzae* ATCC 42149, AO090023000837; *Aspergillus terreus* ATCC 20542, ATEG_05472.1; *Botrytis cinerea* B05.10, BC1G_12307.1; *Chaetomium globosum* CBS 148.51, CHGG_3281.1; *Cladosporium fulvum*, AAF33834.1; *Coccidioides immitis* RS, CIMG_04062.3; *Cochliobolus heterostrophus* C5, CocheC5_1_96332; *Epichloe festucae*, AAQ_73630.1; ***Fusarium* spp**.: *Fusarium graminearum* (*Gibberella zeae* PH-1), FGSG_10825.3; *Fusarium oxysporum* f sp. Lycopersici 4286, FOXG_12759.2; *Fusarium verticillioides* (*Gibberella moniliformis* 7600), FVEG_11500.3; *Nectria haematococca* MPVI (*Fusarium solani*), Nacha2_60819; *Gaeumannomyces graminis* var *tritici*, GGTG_11099.1; *Histoplasma capsulatum* NAm1, HCAG_05565.1; *Leptosphaeria maculans*, XP_003835255.1; *Magnaporthe oryzae* 70-15, MGG_806712.6; *Magnaporthe poae*, MAPG_10080.1; *Mycosphaerella graminicola* (*Zymoseptoria tritici*), Mysgr3_106395; *Neurospora crassa* OR74A, NCU06512.3; *Paracoccidioides brasiliensis* Pb01, PADG_08328.1; *Phaeosphaeria nodorum* SN15, SNOG_04804.1; *Podospora anserina*, Pa_7_8120; *Pyrenophora tritici-repentis* Pt-1C-BFP, PTRG_04270.1; *Sclerotinia sclerotiorum* 1980, SS1G_10919.1; *Trichoderma reesei* QM6a, Trire2_121820; *Tuber melanosporum* Mel28, XP_002837456.1; *Uncinocarpus reesii* 1704, UREG_01910.1; *Verticillium dahliae*, EGY_21633.1; **Basidiomycota**: *Agaricus bisporus* JB137-58, AGABI1DRAFT_113354; *Coprinus cinereus* (*Coprinopsis cinerea*), CC1G_00401.1; *Cryptococcus neoformans* var. neoformans JEC21, CNAG_01890.1; *Hebeloma cylindrosporum* h7, Hebcy2_23860; *Laccaria bicolor*, Lacbi_187396 and Lacbi_183094; *Melamspora laric-populina* 98AG31, MELLADRAFT_79168; *Phanerochaete chrysosporium* RP-78, Phchr1_139663; *Postia placenta* MAD-698, Pospl1_110355; *Puccinia graminis* CRL 75-36-700-3, PGTG_02658.2; *Sporobolomyces roseus*, Sporo1_34369; *Ustilago hordei*, CCF54835.1; *Ustilago maydis* 521, UM00934.1; **Mucoromycota**: *Mucor circinelloides* CBS277.49, Mucci2_142350; *Phycomyces blakesleeanus*, Phybl1_30008 and Phybl1_34694 ; *Rhizopus oryzae*, RO3G_04919.1 and RO3G_16940.1; **Chytridiomycota (Chitrids)**: *Batrachochytrium dendrobatidis* JAMB1, BDEG_06528.1 and BDEG_01881.1. Other organisms are**: Oomycetes**: *Phytophthora infestans*, PITG_01072; *Phytophthora ramorum*, Phyra1_1_72019; *Phytophthora sojae*, Physo1_1_108148. **Green algae**: *Chlamydomonas reinhardtii*, Chlre3_154307; **Red algae**: *Cyanidioschyzon merolae*, CMJ234c; **Brown algae**: *Ectocarpus siliculosus*, CBN73953.1; **Plants spp.**: *Arabidopsis thaliana*, ATMS1 (AT5G17920.2), ATMS2 (AT3G03780.2), ATMS3 (AT5G20980.2); *Glycine max*, AAQ08403; *Hordeum vulgare*, CAJ01714; *Oryza sativa*, ABG22097.1; *Zea mays*, AAL33589**; Bacteria**: *Erwinia carotovora*, Q6DAS2.1; *Escherichia coli*, P25665; *Pseudomonas aeruginosa*, P57703; *Trichodesmium erythraeum* IMS101, Tery_0847 (cyanobacteria).
